# Global transcriptome analysis of *Pseudomonas aeruginosa* NT06 response to potassium chloride, sodium lactate, sodium citrate, and microaerophilic conditions in a fish ecosystem

**DOI:** 10.1093/femsle/fnae043

**Published:** 2024-06-06

**Authors:** Natalia Tomaś, Kamila Myszka, Łukasz Wolko, Wojciech Juzwa

**Affiliations:** Department of Biotechnology and Food Microbiology, Faculty of Food Science and Nutrition, Poznan University of Life Sciences, Wojska Polskiego 48, 60–637 Poznań, Poland; Department of Human Nutrition and Dietotherapy, Faculty of Biological Sciences, University of Zielona Gora, Pałac Kalsk 67, 66–100 Sulechów, Poland; Department of Biotechnology and Food Microbiology, Faculty of Food Science and Nutrition, Poznan University of Life Sciences, Wojska Polskiego 48, 60–637 Poznań, Poland; Department of Biochemistry and Biotechnology, Faculty of Agriculture, Horticulture and Bioengineering, Poznan University of Life Sciences, Dojazd 11, 60–632 Poznań, Poland; Department of Biotechnology and Food Microbiology, Faculty of Food Science and Nutrition, Poznan University of Life Sciences, Wojska Polskiego 48, 60–637 Poznań, Poland

**Keywords:** NaCl alternatives, DEGs, RNA-seq, fish spoilage, virulence, RT-qPCR

## Abstract

*Pseudomonas aeruginosa* is an opportunistic pathogen that recently has been increasingly isolated from foods, especially from minimally processed fish-based products. Those are preserved by the addition of sodium chloride (NaCl) and packaging in a modified atmosphere. However, the current trends of minimizing NaCl content may result in an increased occurrence of *P. aeruginosa*. NaCl can be replaced with potassium chloride (KCl) or sodium salts of organic acids. Herein, we examined the antimicrobial effects of KCl, sodium lactate (NaL), sodium citrate (NaC), and sodium acetate (NaA) against *P. aeruginosa* NT06 isolated from fish. Transcriptome response of cells grown in medium imitating a fish product supplemented with KCl and KCl/NaL/NaC and maintained under microaerophilic conditions was analysed. Flow cytometry analysis showed that treatment with KCl and KCl/NaL/NaC resulted in changed metabolic activity of cells. In response to KCl and KCl/NaL/NaC treatment, genes related to cell maintenance, stress response, quorum sensing, virulence, efflux pump, and metabolism were differentially expressed. Collectively, our results provide an improved understanding of the response of *P. aeruginosa* to NaCl alternative compounds that can be implemented in fish-based products and encourage further exploration of the development of effective methods to protect foods against the *P. aeruginosa*, underestimate foodborne bacteria.

## Teaser

Model studies describing the impact of salt substitutes and microaerophilic conditions used in minimally processed food technology, on the physiology and genetics of *P. aeruginosa*, a food spoilage microorganism.

## Introduction

Given the positive effects of fish in the prevention of cardiovascular diseases, the consumption of minimally processed ready-to-eat seafoods is constantly growing (FAO 2020). However, in fish products subjected to limited physical preservation technologies, bacterial growth is not sufficiently inhibited and can easily overgrow, causing gastrointestinal illness and altering the sensory properties of the product, making it unsuitable for consumption (Pedro and Nunes 2019). In minimal processing of fish, e.g. cold smoking, only steps such as lowering the water activity by salting, dehydrating, smoking, and packaging in a modified atmosphere (MAP) contribute to the preservative effects. The most effective means of lowering the water activity is the addition of sodium chloride (NaCl). However, due to the current trend of minimizing the NaCl content within the products, the occurrence of microbiological spoilers, especially *Pseudomonas aeruginosa*, is common in seafoods (Løvdal 2015). Notably, a number of recent studies showed that *P. aeruginosa* is an emerging pathogen, more frequently isolated from fresh, smoked, salted, or even dried fish (Boss et al. 2016, Benie et al. 2017, Myszka et al. 2019, Algammal et al. 2020, Shahrokhi et al. 2022, Li et al. 2023, Mumbo et al. 2023). They have also natural resistance to a number of antibiotics (Tomaś et al. 2023). The ability of *P. aeruginosa* to grow in chilled foods is attributed to its high adaptability due to the physiological flexibility of the genus. Some studies revealed the role of the two-regulatory component system, which consists of a membrane-enclosed sensor along with a cytoplasmic response regulator that enables survival and growth under refrigerated conditions (Chauhan et al. 2023). Nevertheless, the addition of NaCl to chilled foods is usually sufficient to control *Pseudomonas* spp. proliferation; thus, NaCl reduction is associated with a higher risk of *P. aeruginosa* emergence and persistence. Therefore, NaCl should be replaced with agents that are both effective in hindering *P. aeruginosa* growth and safe for consumers.

The group of potential preservative agents, that can be used in the minimal processing of seafoods are potassium chloride (KCl) and sodium salts of organic acids [e.g. sodium lactate (NaL), sodium citrate (NaC), and sodium acetate (NaA)] (Ghaly et al. 2010, Almli and Hersleth 2013). The possible inhibition mode of those compounds is based on lowering the water activity, similar to NaCl, but also acidification of the microbial cell interior (Shelef 1994). A number of studies have reported the effectiveness of NaCl alternative compounds in retarding the growth of spoilage bacteria in food-model conditions. For example, the work of Pedro and Nunes (2019) revealed that KCl has an equivalent antimicrobial effect to NaCl on bacteria and the 50% replacement of salt did not influence the sensory properties of smoked sea bass. The treatment of Frankfurters and ham by dipping in NaL caused a reduction in *P. aeruginosa* and *P. fluorescens* counts (Oh et al. 2014). Sallam (2007) analyzed the antimicrobial and antioxidant effects of sodium salts applied at 2.5% concentrations in fresh salmon slices. The above compounds retarded the proliferation of spoilage microorganisms belonging to *Pseudomonas* spp., *Enterobacteriaceae* spp., and H_2_S-producing and lactic acid bacteria. Changes in the expression of genes involved in virulence have been also reported after exposure of *P. aeruginosa* to KCl, NaL, and NaC (Tomaś et al. 2023). The recent work of Khayat et al. (2022) showed that NaC inhibited *P. aeruginosa* biofilm formation at sub-MIC concentrations, decreased the production of virulence factors, and lowered bacterial motility. Nevertheless, sublethal doses of antimicrobials, characteristic of minimal processing technology, may result in the development of resistance strategies by microbial cells (Besten et al. 2010). In addition, although MAP was proven to be effective in inhibition of *P. aeruginosa* in fish (Pang et al. 2013), the lowered oxygen concentrations is characteristic for pathogenic nature of *P. aeruginosa* lung infections in cystic fibrosis patients (Alvarez-Ortega and Harwood 2007). Therefore, studies of their overall impact on food-related *P. aeruginosa* strains, need to be conducted in detail to successfully develop preservation technologies. Such investigations should also be carried out at the transcriptomic level, since little is known about food-derived *P. aeruginosa* strains and their adaptation strategies to preservative barriers that are implemented in minimally processed fish-based products. RNA-Seq analysis is an effective method for examining the microbial response to antimicrobials based on transcriptional changes in relation to control conditions (Liu et al. 2017).

The aim of this study was to analyze the physiological response of *P. aeruginosa* NT06 to selected concentrations of KCl/NaL/NaC/NaA and microaerophilic conditions that mimic MAP conditions (Kwan et al. 2013). The inhibitory potential of the above compounds alone and in combination at concentrations that can be used in food matrices was evaluated by the macrodilution method. Flow cytometry analysis was used to assess the cell viability upon treatment with KCl and KCl/NaL/NaC. Comparative transcriptome analysis of total RNA extracted from *P. aeruginosa* NT06 grown in medium imitating a fish product supplemented with KCl and KCl/NaL/NaC and held under microaerophilic conditions was performed. The concentrations of the above compounds used in the study were equal to those routinely applied in food technology (Albarracín et al. 2011, Giese et al. 2019). To validate the transcriptomic analyses, the assessment of the expression levels of selected differentially expressed genes (DEGs) (*napE, ccpR, rhlA, pprB, nalD, pgl*, and *codB*) was conducted. The overall results provide knowledge of the antimicrobial efficacy of NaCl alternatives that can be implemented in minimally processed fish-based products and the response and potential resistance strategies of *P. aeruginosa* cells to those compounds, which is important to develop efficient alternative preservative techniques for the food industry.

## Materials and methods

### Microorganism and culture conditions

In preliminary experiments, pure cultures of *P. aeruginosa* NT06 were isolated from commercially available raw salmon and stored frozen (−80°C) in glycerol stocks. The cells were recovered and cultivated in modified TSB medium imitating a fish product (g/1000 ml of distilled water: 20.0 g of fish peptone, 2.5 g of glucose, 5.0 g of NaCl, and 2.5 g dipotassium phosphate). The cultures were carried out at 4°C for 72 h. The cultures were supplemented with selected concentrations of KCl/NaL/NaC/NaA (Table 1). To generate microaerophilic conditions, the samples were incubated in a jar with the CampyGen Atmosphere Generation System (Oxoid, UK).

**Table 1.** Culture characteristics of *P. aeruginosa* NT06.

Medium variant number	Salt	Concentration (g/l)	Growth inhibition (%)
1	NaCl	5.0	–
2	KCl	2.5	10^e^
3		5.0	73^bcd^
4		6.0	70^cd^
5	NaL	2.5	10^e^
6		5.0	70^cd^
7		6.0	70^cd^
8	NaC	2.5	60^d^
9		5.0	75^bc^
10		6.0	90^a^
11	NaA	2.5	NO^e^
12		5.0	NO^e^
13		6.0	NO^e^
14	KCl/NaL	6.0/6.0	85^ab^
15	KCl/NaL/NaC	6.0/6.0/2.5	99^a^

Percentage inhibition are mean values calculated from three replicates. Statistical differences were calculated using one way ANOVA (*P* < .05). The same letters indicate no statistical differences among experimental conditions. NO—not observed.

### Determination of the *P. aeruginosa* NT06 growth inhibition

The growth inhibition of *P. aeruginosa* after incubation with selected concentrations of KCl/NaL/NaA/NaC was determined by the Koch plate method. Medium variants number 2–15 (Table 1) were inoculated with fresh *P. aeruginosa* culture (4 log CFU/ml) and incubated under microaerophilic conditions as described in 'Microorganism and culture conditions' section. Medium variant number 1 consisted of a control culture and was incubated under aerobic conditions as described by Tryfinopoulou et al. (2002) and Cortés-Sánchez et al. (2023). After 72 h of incubation, 1 ml samples of the cultures and their dilutions were transferred into sterile Petri dishes and poured with ~15 ml of plate count agar medium (Oxoid). The growth inhibition was calculated according to the following equation:


\begin{eqnarray*}
\% \,\,\textit{growth}\,\,\textit{inhibition} = 100 - \left( {\frac{a}{b}*100} \right),
\end{eqnarray*}

where: *a*—colony count on medium supplemented with selected concentrations of KCl/NaL/NaC/NaA; *b*—colony count on control medium (5.0 g NaCl).

### Determination of the *P. aeruginosa* NT06 cell viability and vitality

Samples were examined for cellular metabolic activity and viability using imaging flow cytometer FlowSight™ (Luminex Corp., USA) equipped with three lasers (405, 488, and 642 nm), five fluorescence channels (acquisition by a multichannel CCD camera), and side scatter detector (SSC). Imaging flow cytometry (IFC) protocol was based on Duber et al. (2024). The BacLight™ Redox Sensor™ Green Vitality Kit (Thermo Scientific, Germany) was used, which contains RedoxSensor Green reagent (a fluorogenic redox indicator dye, which is subjected to conversion by microbial reductases involved in electron transport chain) and PI (indicator of cellular integrity). Postacquisition data analysis was performed using the IDEAS software (Luminex Corp.) with Machine Learning (ML) module that improved the discrimination of microbial cells from cellular and noncellular debris as described in detail in the paper of Konieczny et al. (2021).

The resulting classifier (cells_Classifier cells_*vs*_debris) contains a series of 438 differentially weighted features (parameters), which were plotted into histogram and events with values higher than zero represent images that are best represented by microbial cells and thus were gated as subpopulation CELLS.

### Whole transcriptomic analysis (RNA-seq) of *P. aeruginosa* NT06

*Pseudomonas aeruginosa* NT06 was grown under microaerophilic conditions for 72 h at 4°C in modified TSB medium supplemented with KCl and KCl/NaL/NaC. The control culture was performed under aerobic conditions (Tryfinopoulou et al. 2002). Total RNA was isolated using an RNAqueous Kit (Thermo Fisher Scientific, USA) according to the manufacturer’s instructions. Ribosomal RNA was removed using a Ribominus Transcriptome Isolation Kit (Invitrogen, USA). The Collibri™ Stranded RNA Library Prep Kit for Illumina™ and the Collibri™ H/M/R rRNA Depletion Kit (Thermo Fisher Scientific) were used for cDNA library construction. Libraries were quantitatively and qualitatively assessed using a Qubit fluorimeter (Invitrogen) and Bioanalyzer DNA electropherogram (Agilent, USA). Sequencing was performed using MiSeq Reagent Kit v3 (150 cycles) on a MiSeq Illumina sequencer. RNA-Seq data were deposited in the Sequence Read Archive of the National Center for Biotechnology Information (NCBI) database under the following numbers: SRX19555927, SRX19555926, and SRX19555924.

### RNA-seq data analysis

RNA-seq data were analyzed with CLC Genomics Workbench 20.0.4 (Qiagen) software. Raw reads were first filtered (adaptor sequences, low quality bases and short reads were deleted) and then clean reads were mapped to the corresponding genome of *P. aeruginosa* PA01 (Gene Bank accession number: NC_002516). The expression of each coding sequence (CDS) was normalized by the transcripts per million value, which represents a probabilistic estimate of the abundance of gene transcripts across compared samples. CDS annotations marked only with a locus were compared to sequences in the Reference RNA Sequences/Reference Protein databases using the NCBI BLASTN or BLASTX tools, respectively (Sayers et al. 2021). Additionally, the UniProt (The UniProt Consortium 2023) and *Pseudomonas* genome databases (Winsor et al. 2005) were used. Sample distance was analyzed by principal component analysis (PCA). A Venn diagram was constructed to specify the number of common and unique DEGs in relation to the control sample.

### RNA extraction and cDNA synthesis

*Pseudomonas aeruginosa* NT06 cultures were treated with the RNAprotect® Bacteria Reagent (Qiagen, Germany). For RNA isolation and purification, the PureLink™ RNA Mini Kit (Thermo Fisher Scientific) and the PureLink™ DNase Set (Invitrogen) were used according to the manufacturer’s protocols. The RNA extracts were analyzed quantitatively and qualitatively using fluorescence-based Qubit™ XR RNA and Qubit™ IQ RNA Assay Kits (Thermo Fisher Scientific) on a Qubit Fluorometer 4 (Invitrogen). The reverse transcription of 1.0 µg of RNA to cDNA was performed using the High Capacity RNA-to-cDNA Kit (Life Technologies, USA) according to the manufacturer’s protocol.

### RT‒qPCR analyses

The primers (Table S1) for RT‒qPCR analyses were designed using the CLC Genomics Workbench (Qiagen, USA) based on the genome sequence of *P. aeruginosa* PA01. Experiments were performed on a CFX96 system (Bio-Rad, USA) using GoTaq® Master Mix (Promega, Germany). The following cycling conditions were applied: initial denaturation at 95°C for 2 min; 45 cycles of denaturation at 95°C for 15 s, annealing at 55°C, and extension at 72°C for 15 s; followed by a melting curve. The fold changes of analyzed genes were calculated using the 2^−ΔΔCT^ method as described by Schmittgen and Livak (2008).

### Statistical analysis

The statistical analyses were performed in R (R Core Team 2018). The experiments were performed in triplicate. Significant differences (*P* < .05) were established by one–way analysis of variance (ANOVA) followed by Tukey’s *post hoc* test.

## Results and discussion

### Effect of KCl/NaL/NaA/NaC on *P. aeruginosa* growth inhibition

The effects of selected concentrations of salt alternatives, KCl/NaL/NaA/NaC on *P. aeruginosa* NT06 growth were evaluated by the changes in total counts. As presented in Table 1, the proliferation of *P. aeruginosa* NT06 was suppressed by the applied treatments and ranged from ~10% to 99%, according to the concentration used. Only NaA was not effective in decreasing the number of *P. aeruginosa* NT06, regardless of the concentration used. These results are in agreement with the work of Frecti et al. (1979), who reported that the growth of *P. aeruginosa* in protein hydrolysate solutions was not inhibited by the addition of NaA alone. Kamani et al. (2020) reported that the preservation effect of NaA incorporated into chitosan films used for rainbow trout fillet storage was attributable only to a decrease in lipid oxidation.

Among the single compounds tested, the highest inhibition was observed for NaC; 6.0 g/l of NaC decreased the growth of *P. aeruginosa* strains by 90%. There were no statistically significant differences in the inhibitory effects of KCl, and NaL applied at concentrations of 5.0 and 6.0 g/l. The effectiveness of NaL in *P. aeruginosa* growth inhibition shown in the present study was in agreement with the work of Cegielska-Radziejewska and Pikul (2004). In the work, NaL added at 2% to poultry sausage significantly inhibited the development of the psychrotrophic aerobic and lactic acid bacteria and extended their shelf life 4- and 7-fold when packed in air and nitrogen atmosphere, respectively. In contrast to the antimicrobial activity of NaC provided in this study, Khayat et al. (2022) documented that *P. aeruginosa* PAO1 growth was not inhibited by 5% and 6% NaC after 24 h of incubation. These discrepancies may be due to the cell origin; *P. aeruginosa* PA01 was derived from a clinical specimen, while the strains examined here were isolated from food. Notably, antimicrobial resistance traits tend to be more complex in clinical strains than in those from the environment (Bhuiya et al. 2018, Karami et al. 2019).

For medium variants consisting of two or three different NaCl alternatives (numbers 14 and 15), the proliferation of *P. aeruginosa* was significantly arrested to 85%–99%. The highest growth inhibition observed for mixtures of KCl/NaL and KCl/NaL/NaC was in agreement with the work of Kin et al. (2011), in which using a NaA and KCl resulted in extending the shelf life of marinated catfish fillets. The synergistic antimicrobial effect of NaL and NaC was revealed by Long and Phillips (2003), in which the combination of those compounds resulted in a significant reduction of the total counts of *Arcobacter butzleri* NCTC 12481 on chickens.

### Effect of KCl and KCl/NaL/NaC on cell viability and vitality

Stress factors, such as high salt concentrations, are characteristic of the food matrix and can cause the damage to bacterial cells and/or influence on their further viability. By using the flow cytometry, it is feasible to measure the microorganism’s metabolic response to preservative agents or methods (Comas-Riu and Rius 2009). Therefore, the physiological activity of cells after incubation with NaCl (control), KCl (A), and KCl/NaL/NaC (D) was measured using flow cytometric analysis. In order to enhance the resolution of the assay and distinguish between cellular and noncellular debris from microbial cells, IFC was utilized. IFC merges conventional flow cytometry with fluorescence microscopy. The significant novelty and primary impact of this methodology lies in its potential to facilitate the identification of dormant and injured bacterial cells as distinct subpopulations. By combining the statistical power of conventional flow cytometry with the imaging capabilities of microscopy, IFC addresses the limitations of traditional flow cytometry. While traditional flow cytometers only measure fluorescence signal intensities, IFC offers significant advancements by associating fluorescence light parameter data with digitally processed images of analyzed cells. Additionally, IFC provides spatial information regarding the distribution of fluorescent signals within individual cells or particles (Barteneva et al. 2012, Barteneva and Vorobjev 2016). IFC associates fluorescence intensity data with cellular morphology and numerous parameters derived from pixels. IFC employs a charge-coupled device camera (CCD) to capture multiple high-resolution images of every cell in flow, encompassing brightfield, darkfield (SSC), and up to 10 fluorescent markers. These images obtained through IFC are subsequently examined using dedicated software, enabling the extraction of quantitative data for each cell or particle. ML algorithms are integrated to enhance and streamline the classification of the data (Konieczny et al. 2021).

**Figure 1.**
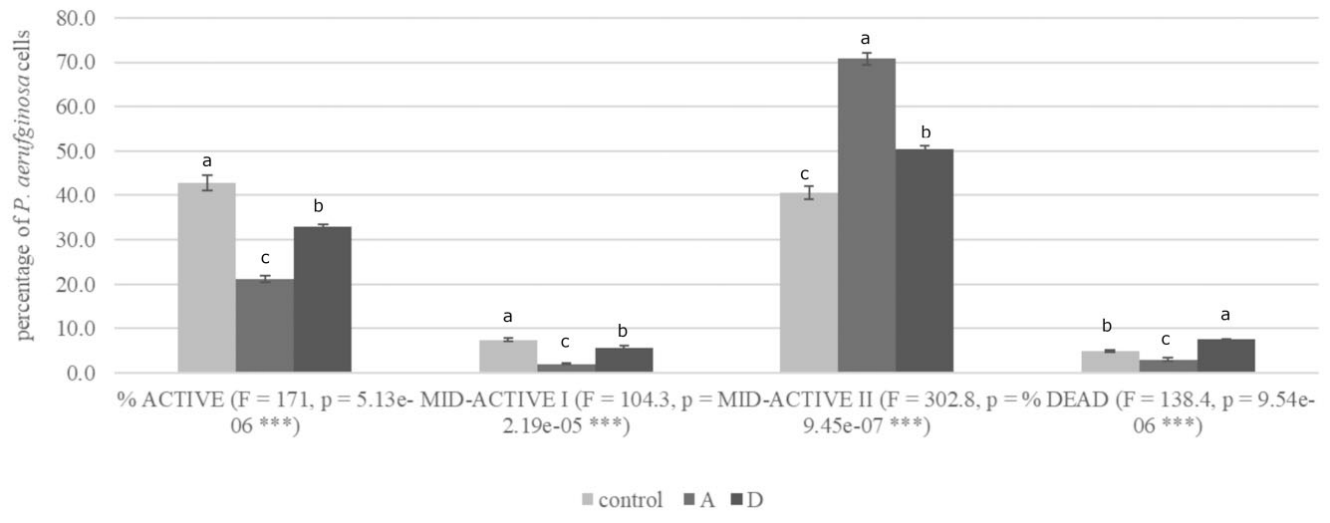
Physiological state of *P. aeruginosa* NT06 cells grown in modified TSB medium supplemented with KCl (A) and KCl/NaL/NaC (D) under microaerophilic conditions and NaCl under aerobic conditions (control).

In our work, ML-based protocol was employed to facilitate and improve the discrimination of microbial cells from cellular and noncellular debris. The functional and structural cellular parameters measured using the fluorescence were redox potential based on the use of RedoxSensorTM Green reagent (Thermo Fisher Scientific) and membrane integrity as measured using propidium iodide (PI) fluorophore, respectively (Table S2). RedoxSensor^TM^ Green reagent is subjected to conversion by microbial reductases, a part of electron transport systems, and following excitation (maximum = 490 nm) emits a green fluorescence (maximum = 520 nm). The intensity of the green fluorescence emission is directly proportional to cellular redox potential level as a result of the activity of cellular reductases, indicating the level of microbial cells metabolic activity. In combination with live/dead discrimination based on red fluorescence intensities from PI the analysis enabled the definition of four subpopulations of microbial cells: dead (demonstrating compromised membranes and low levels of cellular metabolic activity), mid-active I (demonstrating compromised membranes and high levels of cellular metabolic activity—injured cells), mid-active II (demonstrating high membrane integrity but simultaneously low levels of cellular metabolic activity—dormant cells), and active (demonstrating high membrane integrity and high levels of cellular metabolic activity). The discrimination was based on differences in fluorescence signal intensities measured by the specific light detector and expressed as relative fluorescence units. As presented in Fig. 1, sample treated with the KCl had a significantly lower number of active cells (21.2%) and dead cells (3.0%) in relation to salt mixture-treated cells (33.0% and 7.5%, respectively) and control culture (42.8% and 4.9%, respectively). Subpopulations of cells with low levels of metabolic activity and intact low-activity cell membranes (mid-active II) accounted for about 50.6% in the KCl/NaL/NaC-treated population and were significantly higher than in the control sample (40.7%) and simultaneously significantly lower than in the KCl-treated population (70.8%). The inverse relationship was true for the levels of subpopulations of damaged cells (mid-active I). The results proved that the *P. aeruginosa* NT06 population treated with NaCl alternatives can be divided into subpopulations with different physiological activities. Furthermore, the application of more than one NaCl alternative increased the number of injured cells compared to the application of a single compound, and the injured cells switched to a state of intermediate metabolic activity. Gandhi and Shah (2015) showed a similar trend among probiotic bacteria exposed to increased concentrations of NaCl. With increasing concentration of NaCl, the presence of subpopulation with damaged membranes and no esterase activity was shown. Similarly, Sunny-Roberts and Knorr (2008) used the flow cytometry to assess the osmotic stress response among *L. rhamnosus*.

### Overview of the effects of KCl and KCl/NaL/NaC and microaerophilic conditions on the *P. aeruginosa* NT06 transcriptome

Transcriptome sequencing was used to identify the DEGs of *P. aeruginosa* NT06 incubated with NaCl alternatives and under microaerophilic conditions. Libraries were obtained from mRNA isolated from control cultures (medium variant 1), and exposed to KCl, and KCl/NaL/NaC. An overview of the variance of all gene expression evaluated by the PCA (Fig. S1) demonstrated that the applied conditions resulted in distinct *P. aeruginosa* NT06 expression profiles. The results also showed that the gene expression patterns observed for the NaCl alternatives treatment were more similar than those for the control, which can be also a consequence of gas conditions used.

**Figure 2.**
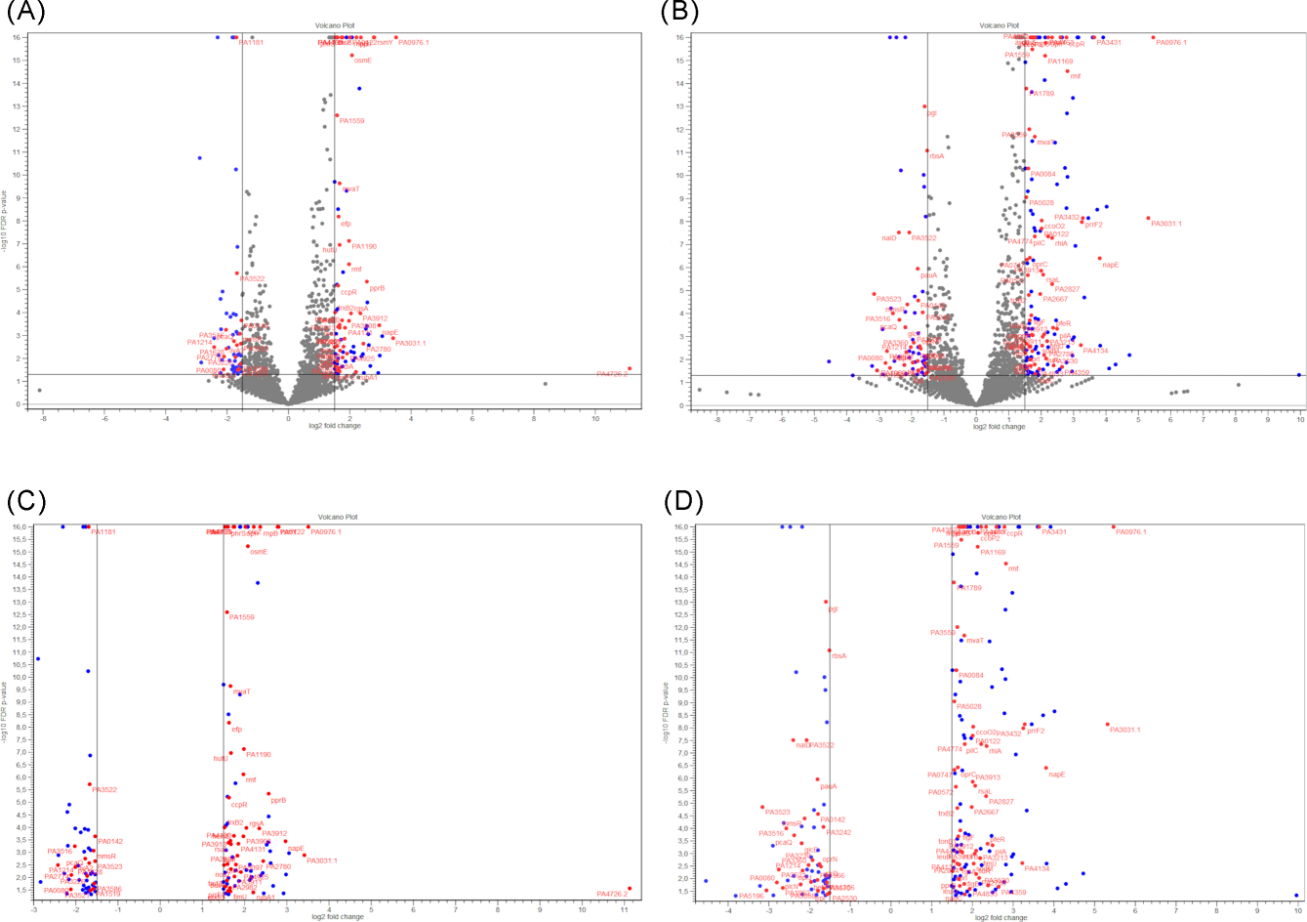
Volcano plots for comparison of transcription profiles of between control and KCl-treated and KCl/NaL/NaC-treated *P. aeruginosa* NT06 cells. (A) Global comparisons of FDR < 0.05 for KCl. (B) Comparisons of FDR < 0.05 and log2-FC ≥ 1.5 for KCl. (C) Global comparisons of FDR < 0.05 for KCl/NaL/NaC. (D) Comparisons of FDR < 0.05 and log2-FC ≥ 1.5 for KCl/NaL/NaC.

Exposure of *P. aeruginosa* NT06 to KCl and microaerophilic conditions resulted in a total of 578 DEGs, among which 345 genes were upregulated and 233 genes were downregulated, while the transcriptomic profile for cells exposed to KCl/NaL/NaC consisted of 738 DEGs (469 upregulated and 269 downregulated) (Fig. 2A and C). According to the Venn diagram (Fig. S2), a total of 354 transcripts were commonly differentially expressed by both treatments. The unique genes that were exclusively divergently expressed accounted for 189 and 347 in the KCl and KCl/NaL/NaC treatments, respectively. To increase confidence in the interpretation of the data on significant changes in bacterial gene expression, dual criteria of *P* ≤ .05 and log2-fold-change (log2-FC) value ≥ 1.5 and ≤ −1.5 were applied.

In total, 152 (26.3% of total) and 218 (29.4% of total) genes satisfied these dual criteria in samples treated with KCl and KCl/NaL/NaC, respectively. KCl treatment induced the upregulation of 94 and downregulation of 58 genes (Fig. 2B), while KCl/NaL/NaC treatment resulted in 144 upregulated and 73 downregulated genes (Fig. 2D). Log2-FC values ranged from 11.03 to −4.57; the average absolute value was 2.06. Because the vast majority of the above DEGs were only described with a locus tag, the putative function was manually assigned and grouped according to the probable role in response to applied treatment. Overall transcriptome results obtained in the following work were in agreement with the paper of Li et al. (2021), who used RNA-Seq analysis to evaluate the effect of increased concentrations of NaCl on *Escherichia coli* BW25113. In the cited work, 1117 DEGs were found and were related to energy metabolism, biofilm formation and virulence. Similarly, Suo et al. (2018) conducted a comparative transcriptome analysis of *Listeria monocytogenes* treated with 4% of NaL and revealed alterations of genes mostly involved in signal transduction, ABC transporters and the phosphotransferase system.

### Functional analysis of DEGs

**Figure 3.**
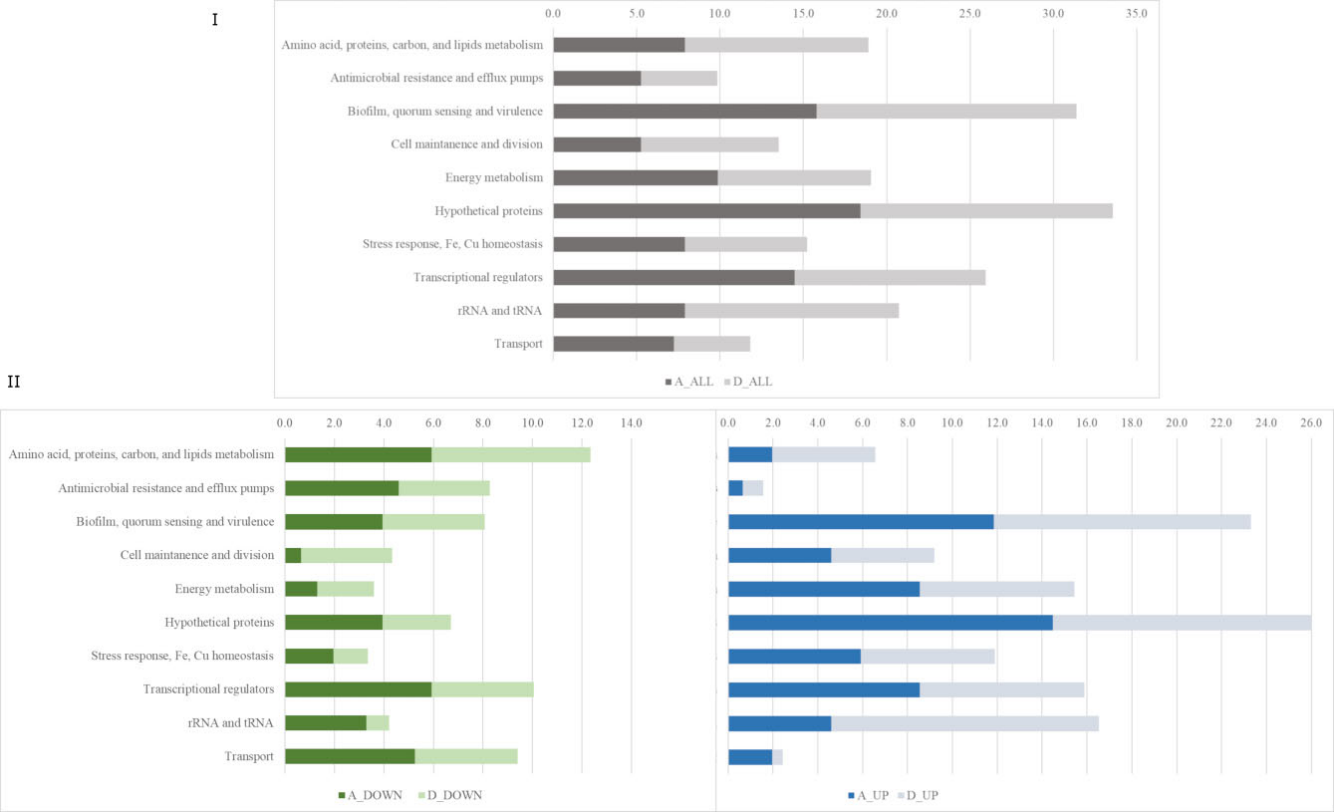
DEGs under KCl (A) and KCl/NaL/NaC (D) treatment of *P. aeruginosa* NT06 cells. (I) Percentage of all DEGs obtained for sample A and D. (II) DEGs divided into down- and upregulated.

**Figure 4.**
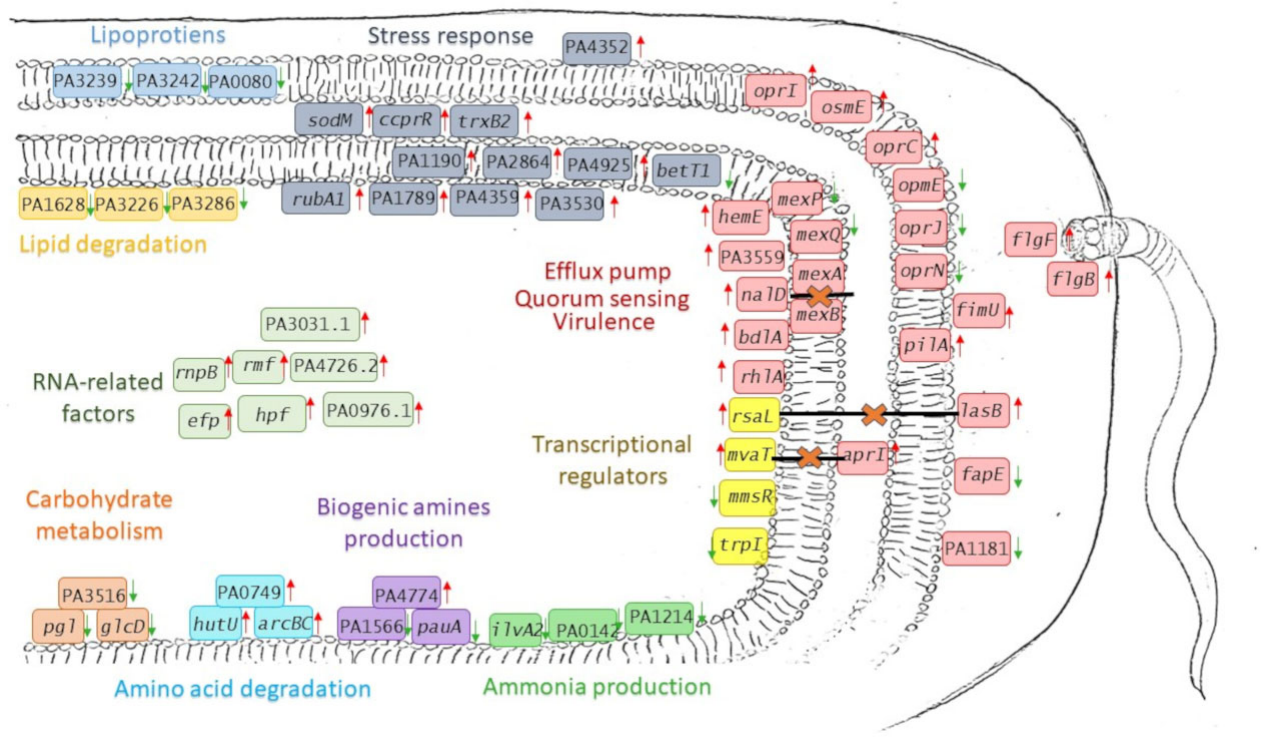
Scheme of *P. aeruginosa* cell response to NaCl alternative compounds and microaerophilic conditions after 72 h incubation at 4°C. Major up- and downregulated DEGs are shown as red and green arrows, respectively. The figure shows DEGs related with ribosome functioning (*rnpB, rmf, efp, hpf*, PA3031.1, PA4726.2, and PA0976.1), redox enzymes (*sodM, ccprR, trxB2*, and *rubA1*), stress response proteins (PA4352, PA1190, PA2864, PA4925, PA1789, PA4359, PA3530, and *betT1*), fimbriae and pili (*pilC, pilA*, and *fimU*), flagellar (*flgB* and *flgF*) and outer membrane proteins (*oprI, osmE, oprE, oprN*, and *oprJ*), lipoproteins (PA0080, PA3239, and PA3242), quorum sensing (*lasB, aprI*, and *rhlA*), virulence and biofilm formation (*hemE*, PA3559, *bdlA, fapE*, and PA1181), transcriptional regulators (*rsaL, mvaT, mmsR*, and *trpI*), efflux pump systems (*mexPQ-opmE* and *nalD*), ammonia production (PA1214, *ilvA2*, and PA0142), biogenic amines production (*pauA*, PA1566, and PA4774), amino acid degradation (*arcB, arcC, hutU*, and PA0747), carbohydrate metabolism (*pgl, glcD*, and PA3516), and lipid degradation (PA3586, PA3226, and PA1628).

Based on functional annotation, DEGs were divided into 10 groups as shown in Fig. 3, however, DEGs that represents ‘energy metabolism’ group was excluded from detailed discussion as they result mostly from oxygen deficiency during *P. aeruginosa* growth. The highest percentage of DEGs was observed for ‘hypothetical proteins’ with unknown, unclassified, or uncharacterized function. Approximately 8% and 13% of DEGs found by samples with KCl and KCl/NaL/NaC, respectively, concerned rRNA and tRNA. The above groups of DEGs are presented in Tables S3 and S4 in the supplementary material. Interestingly, among all RNA-seq results of KCl-treated cells, PA4726.2, which encodes the hypothetical protein p30 that belongs to small ncRNA, had the highest log2-FC value (11.02), but it was absent in KCl/NaL/NaC-treated cells, where the tRNAs PA0976.1 and PA3031.1 encoding the tRNA-Gly and tRNA-Lys, respectively were the most responsive. These RNA fractions are known to be involved in the translation process that generates phenotypic diversity among populations as a consequence of translation errors under stress conditions (Kramer and Farabaugh 2007). According to recent work by Babosan et al. (2022), the modification of RNA expression genes was associated with the *Vibrio cholerae* response to subinhibitory doses of antibiotics, suggesting that differential translation and codon decoding may represent the critical factors involved in the bacterial response to stress. Hayashi et al. (2014) stated that the downregulation of rRNA is one of the mechanisms involved in the response to stimuli by various types of stress, such as hypoxia, energy status, and chemical reagents. The transcriptome results for the rest of the separated groups of DEGs is discussed below and the model of the bacteria response is shown in Fig. 4.

### DEGs classified into the ‘cell maintenance and division’ group

Although not the most numerous, very important DEGs were classified as ‘cell maintenance and division’, which included the genes potentially engaged in cell division and DNA repair thus in cell growth and viability processes (Table S5). In KCl-treated cells, primarily the expression of RNA-related factors, i.e. *rnpB, rmf*, PA4463, and *efp* were induced. RnpB represents an RNA component of RNase P that is essential for tRNA synthesis (Gopalan et al. 2002). Ribosome modulator factor (*rmf*) and PA4463, that is hibernation-promoting factor (*hpf*), are accessory proteins essential for ribosome integrity protection and maintenance of dormant *P. aeruginosa* cells (Franklin et al. 2020). The major role of elongation factor P (*efp*) is to regulate the synthesis of proteins and interact with the translational machinery to reprogram the synthesis of proteins necessary for bacterial virulence and survival against multiple forms of stress (Zou et al. 2011). *Efp* has also been upregulated at low temperatures in *P. psychrophila* MTCC12324 isolated from the Arctic, indicating its function in adapting to low temperatures during cell division and growth (Abraham et al. 2020). In general, the above DEGs represent small regulatory RNAs, that are critical for cell adjustment to physiological conditions, such as cold or starvation (Repoila and Darfeuille 2009). The significant upregulation of PA5028, which probably encodes the *parA* gene, a chromosome segregation ATPase required for precise separation of newly replicated bacterial chromosomes during cell division was observed (Kawalek et al. 2023) as well as O6-methylguanine-DNA methyltransferase, a DNA repair enzyme that provides chemoresistance to alkylating agents (Yu et al. 2020).

### DEGs classified into the ‘stress response, and Fu, Cu homeostasis’ group

Microaerophilic conditions and NaCl alternative compounds also resulted in significant upregulation of *P. aeruginosa* NT06 genes involved in ‘stress response, and Fu, Cu homeostasis’, whose percentage was at the level of 8% in both samples. In KCl-treated cells, the highest upregulation was observed for *rubA1* and PA1190, which encode rubredoxin 1 and Yip1 domain-containing protein, respectively (Table S6). In KCl/NaL/NaC-treated cells, the highest upregulation was observed for PA2827 and *sodM*. The above DEGs represent oxidative stress response systems. *Pseudomonas aeruginosa* cells were also enriched with the *ccpR, trxB2*, and PA2864, providing protection against toxic peroxides. Indeed, rubredoxin and thioredoxin are cellular systems known from anaerobic and microaerophilic bacteria that catalyze a spectrum of redox reactions in the cell as the key response to oxidative stress (Hagelueken et al. 2007, Serrano et al. 2007). Additionally, enzymes such as superoxide dismutases, peroxidases, and catalases are used by bacteria to protect themselves against reactive forms of oxygen (Imlay 2019). Interestingly, transcripts of genes related to osmotic stress in KCl-treated cells (PA4925 and PA4739) were increased, while those in KCl/NaL/NaC (PA3888 and *betT1*) were decreased. Treatment with KCl/NaL/NaC resulted in a higher increase in the mRNA levels of PA4352 and PA1789, which are universal stress proteins, essential for the maintenance of cell growth, both under anaerobic conditions and during other stress factors, e.g. oxidative stress (Boes et al. 2006). Reactive oxygen species are generated by organic acids and result in the DNA damage response in bacteria (Lund et al. 2020). Expression changes in PA4359 (ferrous ion transporter), and PA3530 (ferredoxin Bfd) indicated iron starvation stress, especially for cells exposed to KCl/NaL/NaC (Wang et al. 2007).

### DEGs classified into the ‘biofilm, quorum sensing, and virulence’ group

The transcriptomes were considerably enriched in ‘biofilm, quorum sensing, and virulence’ genes, which were the second most abundant group of DEGs in both samples (Table S7). This was not surprising, because *P. aeruginosa* is an opportunistic pathogen, as it produces a large repertoire of virulence determinants affecting its pathogenicity. The pathogenic nature of *P. aeruginosa* is mostly determined by its movement, adhesion, and colonization ability *via* the production of flagella, pili, and lipopolysaccharides (LPS; Liao et al. 2022). The transcriptome results of KCl/NaL/NaC-treated cells showed considerable upregulation of DEGs implicated in pilins, adhesins, and flagellum, that mediate twitching motility, biofilm maturation, surface adhesion, and virulence (Marko et al. 2018), such as *pilA, pilC, fimU, flgB*, and *flgF*, while in KCl-treated cells lipoprotein PA1041, a biofilm dispersion locus *bdlA*, and pilus assembly protein *tadZ* were the most responsive. However, the expression of PA1356, which promotes colonization, adhesion and biofilm formation was decreased, and simultaneously, PA2780, which is involved in the negative regulation of cell motility and biofilm formation, was upregulated. Such an inconsistent outcome was also observed in the work of Suo et al. (2018), where although flagellar synthesis in *L. monocytogenes* cells was inhibited by NaL, the overall virulence capacity was induced.

*Pseudomonas aeruginosa* cells easily form biofilms, which are complex aggregates of bacteria surrounded by a self-generated matrix consisting of exopolysaccharides (EPS) and LPS (Thi et al. 2020). The transcriptome results of cells incubated with NaCl alternatives showed the induced expression of PA3559, which encodes an enzyme UDP-glucose 6-dehydrogenase involved in the synthesis of EPS and LPS, critical for bacterial virulence (Hung et al. 2007). Additionally, *rhlA*, which participates in rhamnolipid biosynthesis, was also upregulated by NaCl alternative compounds. Simultaneously, the expression of DEGs responsible for biofilm formation and stabilization (PA1952 (*fapE*), PA1181) was repressed. The upregulation of *tonB1*, which is involved in iron import into cells, and *oprC*, the outer membrane porin involved in siderophore transport confirmed the iron-limited conditions. In the transcriptome obtained from KCl-treated cells, increased expression of *hemE* involved in heme biosynthesis required for pathogenesis was observed. According to a review by Kang and Kirienko (2018), iron acquisition systems are crucial for biofilm formation in *P. aeruginosa*.

A large group of DEGs, both up- (*oprI, osmE*, and *oprC*) and downregulated (PA0080, PA3239, and PA3242) concerned lipoproteins and outer membrane proteins. According to the studies conducted by Munguia et al. (2017), *P. aeruginosa* with deleted PA3239 had increased membrane permeability and sensitivity to detergents. Moreover, the PA3242 mutant showed inhibited growth, greater susceptibility to antibiotics and attenuated virulence (Liang et al. 2016). In KCl/NaL/NaC-treated cells, higher upregulation of type VI secretion system-related genes was observed. The major role of the TssC1 protein (PA0084) is an essential component of this secretion system that promotes antibiotic resistance in biofilms (Zhang et al. 2011). In KCl-treated cells, PA2774 encoding a toxic effector protein was repressed, while in the case of the salt mixture, the expression of PA3360 was the most decreased. Mostly, genes involved in toxins and enzymes, including PA0122, PA1169, PA3908, PA4833, and PA0572 *lasB* and *aprI* were induced. Decreased expression concerned the protein involved in the transport of hemolysin (PA3360) in the case of KCl/NaL/NaC-treated cells and toxic effector protein (PA2774) in the case of KCl treatment.

### DEGs encoding ‘transcriptional regulators’

Genes classified as ‘transcriptional regulators’ were highly responsive to the applied *P. aeruginosa* growth conditions (Table S8). In general, treatment with NaCl alternative compounds induced changes in transcriptional regulatory traits with ~8% of the upregulated genes and a 5% of the downregulated genes. Many of the induced DEGs (*rsmY, pprB, crcZ*, and *phrS*) encoded small regulatory RNAs associated with quorum sensing that were significantly upregulated in biofilm structures in relation to planktonic cells (Dötsch et al. 2012). However, at the same time, negative regulators of quorum sensing and virulence, such as *rgsA, rsaL*, PA2667 and *mvaT*, showed considerable upregulation. The role of RgsA in the oxidative stress response was also shown (Hou et al. 2021). Overexpression of *rasL*, which is an antisense of *lasR*, resulted in reduced *lasB* expression and decreased elastase activity (de Kievit et al. 1999). Moreover, MvaT induces the expression of *ptxS*, a repressor of exotoxin A, one of the major virulence factors in *P. aeruginosa* (Westfall et al. 2004). In addition, in KCl/NaL/NaC-treated cells, the genes involved in iron homeostasis (*prrF2* and *pfeR*) were induced. DEGs with decreased expression were associated with transcriptional regulation of amino acid biosynthesis (*trpI* and *mmsR*) and aromatic compound degradation (*liuR* and *pcaQ*).

### DEGs encoding ‘antimicrobial resistance and efflux pumps’

In addition to alteration in virulence traits, NaCl alternatives also impaired the expression of DEGs classified as ‘antimicrobial resistance and efflux pumps’ (Table S9), which lists the genes associated with bacterial resistance to a broad spectrum of antimicrobial agents, also previously identified in transcriptome of *P. aeruginosa* NT06 (Tomaś et al. 2023). They represented ~5% of all DEGs of both analysed samples and were mostly downregulated. Accordingly, in *P. aeruginosa* NT06 cells the downregulation of PA3523–PA3522–PA3521 operon was observed, which encodes the MexPQ-OpmE efflux pump (EP). In addition, cells treated with KCl/NaL/NaC showed considerable downregulation of *oprN* and *oprJ*, the outer membrane subunits of MexEF-OprN and MexCD-OprJ, respectively. Moreover, the upregulation of *nalD*, repressor of MexAB-OprM was observed. EPs are mechanisms responsible for extruding the antimicrobials, mostly antibiotics, outside the cells, as well as responding to stress factors such as reactive oxygen and reactive nitrate or antiseptics and detergents, while also affecting the translation process (Poole 2008). In contrast to the results obtained in this study, those stressors mostly induce overexpression of EPs (Zwaid and Al-Dahmoshi 2022). Because MexPQ-OpmE has been shown to have a major role in the export of macrolides, fluoroquinolones, and other antimicrobials (Mima et al. 2005, Zgurskaya et al. 2022), it can be assumed that applied treatment may affect the antimicrobial resistance profile of *P. aeruginosa* (Tomaś et al. 2023). In both analysed samples, the only elevated expression was observed for PA1559, which encodes the cationic peptide resistance protein CrpA, responsible for adaptive resistance to polymyxins and other antimicrobial peptides (CAP) acting as host innate defense system (Gutu et al. 2015).

### DEGs involved in ‘transport’

Similar to the previous group of DEGs, which is a subgroup of general transport processes, decreased expression upon treatment with NaCl alternative compounds was mostly detected (Table S10). Most of the identified DEGs belong to MFS and ABC-type transporter family proteins. MFS family proteins allow for both: passive transport (from higher to lower concentrations) and, more often, active transport (against the concentration gradient using the energy from ATP hydrolysis). Thus, the lowered expression of MFS transporters may be a consequence of the collapse of the proton motive force of the cell membrane (Stephen et al. 2023). Furthermore, downregulation of *rbsA* associated with ABC-mediated transport of d-ribose may result in altered ribosome quality and mRNA translation processes (Lu et al. 2022). In KCl/NaL/NaC-treated cells, there was significantly decreased expression of PA3670, another component of the ABC transport system. According to the work of Coleman et al. (2020), the product encoded by PA3670 implicated in susceptibility to antibiotics was downregulated in swarming *P. aeruginosa* cells. Interestingly, in KCl-treated cells, there was decreased expression of PA1519, which is a solute carrier protein, known for osmoprotective functions (Jordan et al. 2008). The only upregulated gene identified in the salt mixture was PA3213, encoding an ABC-type transporter that maintains outer membrane lipid asymmetry (Ekiert et al. 2022). In KCl-treated cells, PA5097 (amino acid permease), PA2982 (transport of siderophores), and *opdO* (pyroglutamate porin), were upregulated, indicating the nutrient-limited conditions of *P. aeruginosa* cell growth (Tamber et al. 2006).

### DEGs involved in ‘amino acid, protein, carbon, and lipid metabolism’

Nitrogen and carbon compounds or lipids are essential nutrients for heterotrophic bacterial growth, which refers to supplying energy and biosynthesis of cell components (Kim and Gadd 2008). Therefore, microbial spoilage of seafood mostly refers to organic compound degradation (Comi 2017). Transcriptomic results showed that most of the DEGs related to metabolism were downregulated, which indicates the inhibitory effect of applied treatments on the spoilage potential of *P. aeruginosa* (Table S11).

Both growth conditions decreased the expression of DEGs involved in nitrogen compound degradation, whose activity results in ammonia formation (PA1214, *ilvA2*, and PA0142). In KCl/NaL/NaC-treated cells, there was significant repression of PA2530, PA5196, and *plcN*, which encode metalloprotease TldD/E, ATP-dependent zinc protease, and nonhemolytic phospholipase, respectively, with a role in protein degradation and pathogenesis (Newman et al. 2017). During fish spoilage caused by microbial activity, the production of biogenic amines via decarboxylation of amino acids also occurs. High amounts of biogenic amines in seafood influence food-borne illness and change sensory properties (Wunderlichová et al. 2014, Biji et al. 2016). In this study, DEGs associated with putrescine and cadaverine formation (*pauA*, PA1566) were considerably downregulated, and PA4774 encoding an enzyme that catalyzes the spermidine synthesis from putrescine was upregulated upon treatment with KCl/NaL/NaC. Higher upregulation of DEGs associated with amino acid degradation (*arcB, arcC, hutU*, and PA0747) in the salt mixture than in KCl-treated cells was noticed. In accordance with this study, overexpression of *arcC* was related to oxygen depletion as well as other stress factors (Larsen et al. 2016). Moreover, the gene cluster *leuB–leuD*, which is involved in the biosynthesis of leucine was also upregulated in KCl/NaL/NaC-treated cells. Based on studies performed by Matsumoto et al. (2011), the synthetic operon consisting of *leuB* and *hisC* genes is an essential element in cell growth during starvation.

In KCl/NaL/NaC-treated cells, induced expression of *lldD* encoding an l-lactate dehydrogenase, an essential enzyme for lactate degradation, was also observed (Wang et al. 2018). In KCl-treated cells, genes associated with lipid degradation, e.g. PA3586 and PA3226, encoding hydrolases, and PA1628, encoding dehydrogenase, were downregulated.

### Validation of RNA-Seq by RT–qPCR

**Figure 5.**
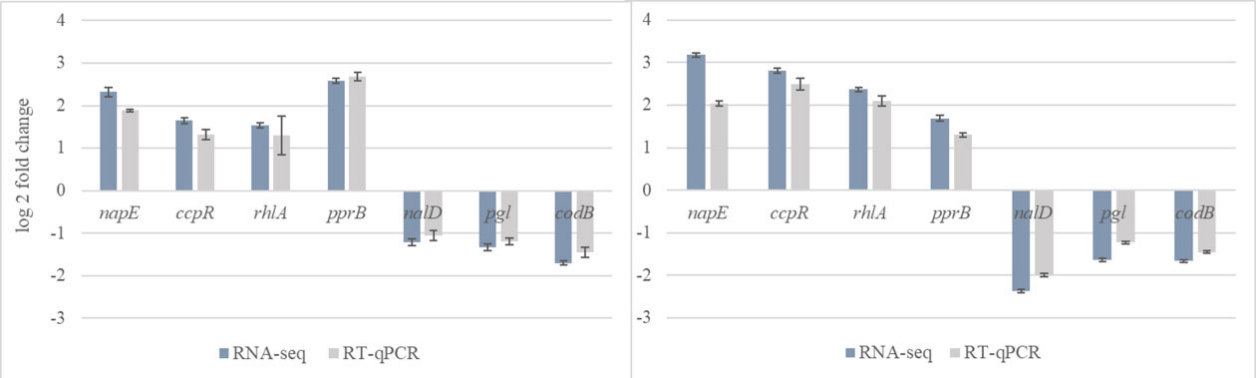
The transcript levels of selected genes of *P. aeruginosa* NT06 exposed to KCl (probe A) and KCl/NaL/NaC (probe D) and microaerophilic conditions evaluated by RNA-seq and RT-qPCR analyses

The transcriptome sequencing results were validated by performing RT–qPCR analyses of the selected genes from control and from KCl-, KCl/NaL/NaC-treated *P. aeruginosa* NT06 cells. As shown in Fig. 5, although there was a minor difference in the fold change values, all the selected DEGs showed consistent expression patterns between the RNA-Seq and RT–qPCR results.

## Conclusions

In conclusion, this study provided the first time the knowledge about *P. aeruginosa* isolated from fish and its physiological and transcriptome response to microaerophilic conditions and NaCl alternative compounds: KCl, NaL, and NaC. Significantly altered genes and cell metabolic states were identified and provided a promising introduction to further studies on the potential application of NaCl alternatives in minimally processed fish-based products. In general, it can be concluded that KCl/NaL/NaC induce higher stress than KCl, causing *P. aeruginosa* cells to respond similarly to their response under starvation conditions. However, the differential expression of a large group of transcripts encoding hypothetical proteins or proteins with unknown functions suggests the need to investigate their role in the salt stress response in future research.

## Supplementary Material

fnae043_Supplemental_File
