# Supplementary material for: Global transcriptome analysis of Pseudomonas aeruginosa NT06 response to potassium chloride, sodium lactate, sodium citrate, and microaerophilic conditions in a fish ecosystem
Source: FEMS Microbiol Lett. 2024 Jun 6;371:fnae043. doi: 10.1093/femsle/fnae043 (PMC11538994; doi:10.1093/femsle/fnae043)
Supplement: fnae043_Supplemental_File [file fnae043_supplemental_file.docx]

**SUPPLEMENTARY MATERIAL**


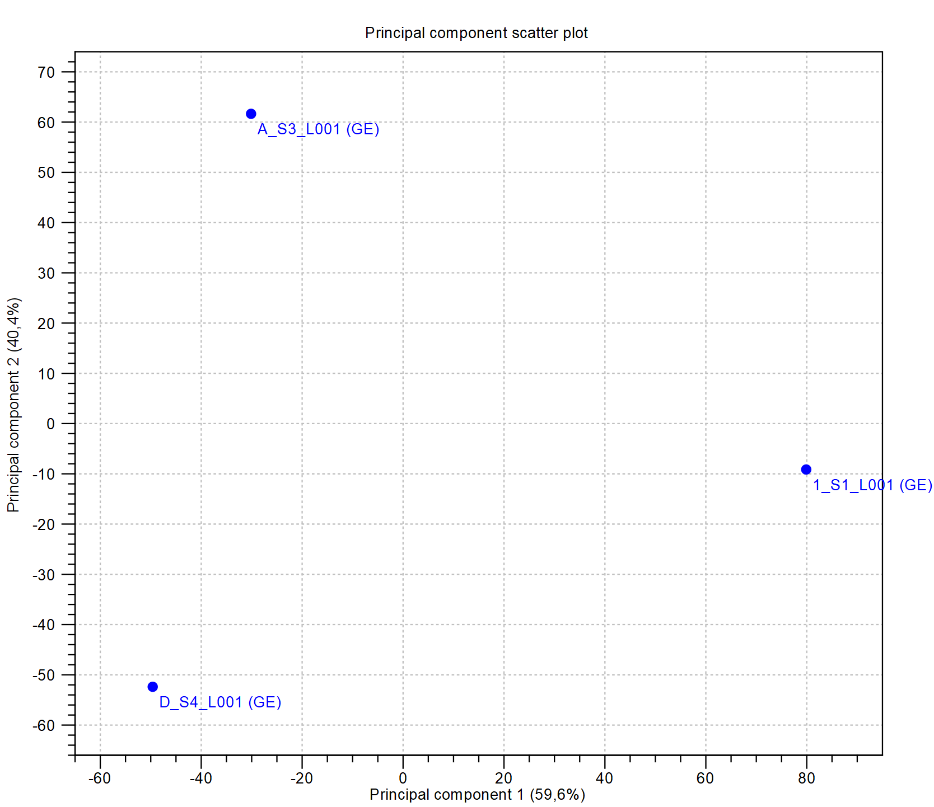


Fig. S1. Principal component analysis of transcriptome data obtained from *P. aeruginosa* NT06 cells grown in modified TSB medium at aerobic conditions (1), and supplemented with KCl (A), and KCl/NaL/NaC (D) under microaerophilic conditions.


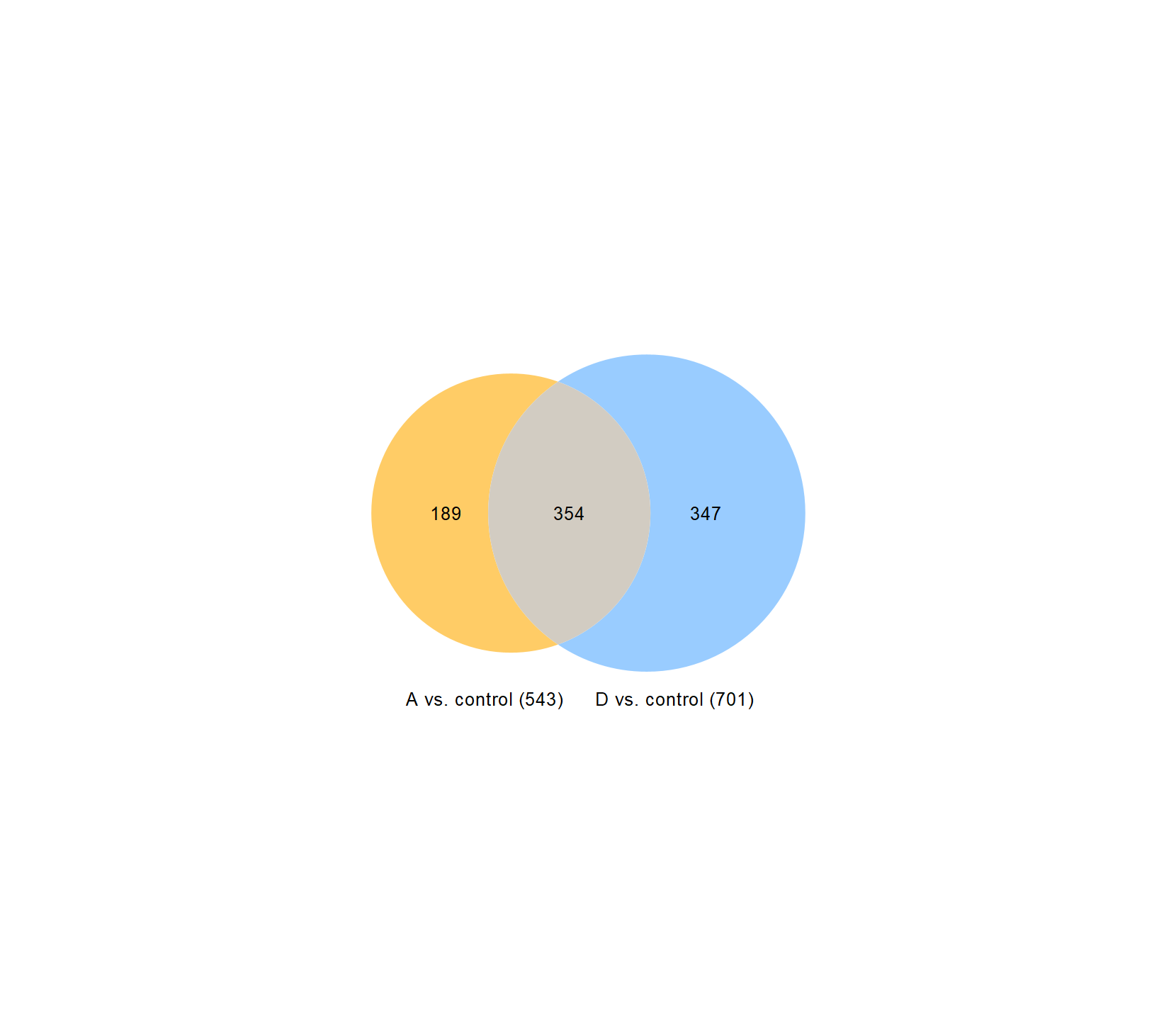


Fis. S2. Venn diagram of overall transcriptome analysis of *P. aeruginosa* NT06 after treatment with KCl/ (A) and KCl/NaL/NaC (D). The sum of the numbers in each circle represents the number of unique DEGs of each probe, and the overlapping portion of the circle indicates the number of common DEGs for both probes

Table S1. List of genes evaluated in the RT-qPCR analyses

| Gene name | Sequence (5’-3’)  Fwd  Rev | Tm  (^o^C) | Size  (bp) |
| --- | --- | --- | --- |
| *16S rRNA* | GGAGACTGCCGGTGACAAACT  TGTAGCCCAGGCCGTAAGG | 56 | 75 |
| *napE* | AGCCAGGCTCTTCCTGTTTC  GGCCAGTAGCTGGTACATCC | 60 | 103 |
| *ccpR* | TCGACGCACAGCAGAAGAAA  TACTCGTCGCTCTGGGTCTT | 60 | 187 |
| *rhlA* | AAATGCACGTGGCTCTGGAT  AACGAGACCGTCGGCAAATA | 60 | 176 |
| *pprB* | TACGAGGTACACGGCAACAG  CCCAGCTCGTAGGCTATCTG | 60 | 197 |
| *nalD* | AACGTTGCGAATTCACCGAG  CGCAACCAGTCGTTGAACAG | 60 | 196 |
| *pgl* | CAGGGGTACCAATGGATCGTC  GATCCAGGGCCAGTCCAAGC | 60 | 117 |
| *codB* | CACATGCTGGCGGAAAAGAA  GTCCGCAGACAGCAACTACA | 60 | 111 |

Table S2. The functional and structural cellular parameters used for machine learning (ML) protocol.

| **Feature** | **weight%** |
| --- | --- |
| Diameter_Object(M02,RSG,Tight) | 0.75 |
| Modulation_M02_RSG | 0.75 |
| Width_Object(M02,RSG,Tight) | 0.74 |
| Length_Object(M02,RSG,Tight) | 0.74 |
| Perimeter_Object(M02,RSG,Tight) | 0.74 |
| Height_Object(M02,RSG,Tight) | 0.74 |
| Thickness Max_Object(M02,RSG,Tight) | 0.73 |
| Thickness Min_Object(M02,RSG,Tight) | 0.72 |
| Area_Skeleton(Object(M02,RSG,Tight),RSG,Thin) | 0.68 |
| Area_Object(M02,RSG,Tight) | 0.67 |
| Modulation_Morphology(M02,RSG)_RSG | 0.66 |
| Elongatedness_Object(M02,RSG,Tight) | 0.64 |
| Mean Pixel_Object(M02,RSG,Tight)_RSG | 0.61 |
| Mean Pixel_M02_RSG | 0.61 |
| Mean Pixel_Valley(M02,RSG,3)_RSG | 0.58 |
| Width_M02 | 0.58 |
| Min Pixel_Skeleton(M02,RSG,Thin)_RSG | 0.57 |
| Thickness Max_M02 | 0.57 |
| Contrast_Morphology(M02,RSG)_RSG | 0.57 |
| Minor Axis_M02 | 0.57 |
| Contrast_M02_RSG | 0.56 |
| Min Pixel_Skeleton(Object(M02,RSG,Tight),RSG,Thin)_RSG | 0.56 |
| Intensity_Valley(M02,RSG,3)_RSG | 0.55 |
| Intensity_Valley(Object(M02,RSG,Tight),RSG,3)_RSG | 0.55 |
| Circularity_M02 | 0.55 |
| Mean Pixel_Skeleton(M02,RSG,Thin)_RSG | 0.55 |
| Bright Detail Intensity R3_MC_RSG | 0.54 |
| Median Pixel_M02_RSG | 0.54 |
| Intensity_MC_RSG | 0.54 |
| Thickness Min_M02 | 0.54 |
| Diameter_M02 | 0.54 |
| Intensity_Valley(Morphology(M02,RSG),RSG,3)_RSG | 0.54 |
| Perimeter_M02 | 0.54 |
| Mean Pixel_Valley(Object(M02,RSG,Tight),RSG,3)_RSG | 0.54 |
| Mean Pixel_Skeleton(Object(M02,RSG,Tight),RSG,Thin)_RSG | 0.54 |
| Area_M02 | 0.53 |
| Mean Pixel_Skeleton(Morphology(M02,RSG),RSG,Thin)_RSG | 0.52 |
| Contrast_Object(M02,RSG,Tight)_RSG | 0.52 |
| Min Pixel_Skeleton(Morphology(M02,RSG),RSG,Thin)_RSG | 0.52 |
| H Entropy Mean_M02_RSG_1 | 0.52 |
| Mean Pixel_Valley(Morphology(M02,RSG),RSG,3)_RSG | 0.51 |
| Std Dev_M02_RSG | 0.51 |
| Shape Ratio_Object(M02,RSG,Tight) | 0.51 |
| Aspect Ratio_Skeleton(M02,RSG,Thin) | 0.5 |
| Aspect Ratio Intensity_Skeleton(Morphology(M02,RSG),RSG,Thin)_RSG | 0.5 |
| Aspect Ratio_M02 | 0.5 |
| Aspect Ratio_Morphology(M02,RSG) | 0.5 |
| Mean Pixel_Morphology(M02,RSG)_RSG | 0.5 |
| Aspect Ratio Intensity_M02_RSG | 0.49 |
| Lobe Count_Morphology(M02,RSG)_RSG | 0.49 |
| Height_M02 | 0.49 |
| Aspect Ratio_Skeleton(Morphology(M02,RSG),RSG,Thin) | 0.49 |
| Gradient RMS_M02_RSG | 0.48 |
| Max Pixel_Skeleton(Object(M02,RSG,Tight),RSG,Thin)_RSG | 0.48 |
| Max Pixel_Valley(Object(M02,RSG,Tight),RSG,3)_RSG | 0.48 |
| Max Pixel_Valley(Morphology(M02,RSG),RSG,3)_RSG | 0.48 |
| Perimeter_Morphology(M02,RSG) | 0.48 |
| Max Pixel_MC_RSG | 0.48 |
| Max Pixel_Valley(M02,RSG,3)_RSG | 0.48 |
| Raw Max Pixel_MC_RSG | 0.48 |
| Lobe Count_Skeleton(Morphology(M02,RSG),RSG,Thin)_RSG | 0.48 |
| Compactness_Object(M02,RSG,Tight)_RSG | 0.48 |
| Intensity_Skeleton(Morphology(M02,RSG),RSG,Thin)_RSG | 0.48 |
| Max Pixel_Skeleton(Morphology(M02,RSG),RSG,Thin)_RSG | 0.48 |
| Max Pixel_Skeleton(M02,RSG,Thin)_RSG | 0.48 |
| Major Axis_M02 | 0.48 |
| H Entropy Mean_M02_RSG_3 | 0.47 |
| Mean Pixel_Threshold(M02,RSG,30%)_RSG | 0.47 |
| Minor Axis_Morphology(M02,RSG) | 0.47 |
| Gradient Max_M02_RSG | 0.47 |
| H Contrast Mean_M02_RSG_3 | 0.46 |
| H Correlation Mean_M02_RSG_1 | 0.46 |
| Minor Axis Intensity_M02_RSG | 0.46 |
| Diameter_Morphology(M02,RSG) | 0.46 |
| Compactness_Morphology(M02,RSG)_RSG | 0.46 |
| Aspect Ratio Intensity_Skeleton(M02,RSG,Thin)_RSG | 0.46 |
| Lobe Count_Skeleton(M02,RSG,Thin)_RSG | 0.46 |
| Std Dev_Morphology(M02,RSG)_RSG | 0.45 |
| H Variance Mean_M02_RSG_3 | 0.45 |
| Major Axis_Morphology(M02,RSG) | 0.44 |
| H Correlation Mean_M02_RSG_3 | -0.44 |
| Thickness Min_Morphology(M02,RSG) | 0.44 |
| Height_Morphology(M02,RSG) | 0.44 |
| Intensity_Skeleton(Object(M02,RSG,Tight),RSG,Thin)_RSG | 0.44 |
| Shape Ratio_M02 | 0.44 |
| H Variance Mean_M02_RSG_1 | 0.43 |
| Circularity_Morphology(M02,RSG) | 0.43 |
| Width_Morphology(M02,RSG) | 0.43 |
| Intensity_Skeleton(M02,RSG,Thin)_RSG | 0.43 |
| Elongatedness_Morphology(M02,RSG) | 0.43 |
| Area_Skeleton(Morphology(M02,RSG),RSG,Thin) | 0.42 |
| Thickness Max_Morphology(M02,RSG) | 0.41 |
| Area_Threshold(M02,RSG,30%) | 0.4 |
| H Homogeneity Mean_M02_RSG_3 | 0.4 |
| Area_Morphology(M02,RSG) | 0.4 |
| Std Dev_M01_BF | -0.4 |
| Length_M02 | 0.4 |
| Raw Max Pixel_MC_PI | 0.4 |
| Max Pixel_MC_PI | 0.4 |
| Std Dev_Morphology(M01,BF)_BF | -0.4 |
| Length_Morphology(M02,RSG) | 0.39 |
| Major Axis Intensity_M02_RSG | 0.38 |
| Bright Detail Intensity R3_MC_PI | 0.38 |
| Bright Detail Intensity R7_MC_RSG | 0.37 |
| Area_MC | 0.37 |
| Contrast_M01_BF | -0.35 |
| Contrast_Morphology(M01,BF)_BF | -0.35 |
| H Homogeneity Mean_M02_RSG_1 | 0.34 |
| Symmetry 3_Skeleton(M02,RSG,Thin)_RSG | 0.33 |
| Min Pixel_Valley(Object(M02,RSG,Tight),RSG,3)_RSG | -0.33 |
| Shape Ratio_Morphology(M02,RSG) | 0.33 |
| Symmetry 4_Skeleton(M02,RSG,Thin)_RSG | 0.32 |
| Lobe Count_Skeleton(M02,RSG,Thick)_RSG | 0.31 |
| Intensity_MC_PI | 0.31 |
| Compactness_M02_RSG | 0.31 |
| Area_Skeleton(M02,RSG,Thin) | 0.31 |
| Std Dev_Object(M01,BF,Tight)_BF | -0.3 |
| H Correlation Mean_M01_BF_1 | -0.3 |
| Symmetry 4_Skeleton(Morphology(M02,RSG),RSG,Thin)_RSG | 0.3 |
| H Variance Std_M02_RSG_3 | 0.3 |
| Mean Pixel_Morphology(M01,BF)_BF | 0.29 |
| Mean Pixel_M01_BF | 0.29 |
| Intensity_Skeleton(Morphology(M01,BF),BF,Thin)_BF | 0.28 |
| Raw Min Pixel_MC_PI | -0.28 |
| Modulation_M01_BF | -0.28 |
| Raw Min Pixel_MC_BF | 0.28 |
| Mean Pixel_Skeleton(Morphology(M01,BF),BF,Thin)_BF | 0.28 |
| Mean Pixel_Skeleton(M01,BF,Thin)_BF | 0.28 |
| Modulation_Morphology(M01,BF)_BF | -0.28 |
| Intensity_Skeleton(M01,BF,Thin)_BF | 0.28 |
| Symmetry 4_Morphology(M02,RSG)_RSG | 0.27 |
| Intensity_MC_BF | 0.27 |
| H Contrast Std_M02_RSG_3 | 0.27 |
| Min Pixel_Skeleton(Morphology(M01,BF),BF,Thin)_BF | 0.26 |
| Mean Pixel_Skeleton(Object(M01,BF,Tight),BF,Thin)_BF | 0.26 |
| Min Pixel_Skeleton(M01,BF,Thin)_BF | 0.26 |
| Symmetry 2_Skeleton(Morphology(M02,RSG),RSG,Thin)_RSG | 0.26 |
| Modulation_Object(M01,BF,Tight)_BF | -0.26 |
| Elongatedness_M02 | 0.25 |
| Min Pixel_Valley(Morphology(M01,BF),BF,3)_BF | 0.25 |
| Mean Pixel_Valley(Morphology(M01,BF),BF,3)_BF | 0.25 |
| Min Pixel_Skeleton(Object(M01,BF,Tight),BF,Thin)_BF | 0.25 |
| Intensity_Skeleton(Object(M01,BF,Tight),BF,Thin)_BF | 0.25 |
| Min Pixel_Valley(Object(M01,BF,Tight),BF,3)_BF | 0.25 |
| H Energy Mean_M02_RSG_3 | 0.25 |
| Min Pixel_Valley(M01,BF,3)_BF | 0.25 |
| Symmetry 2_Morphology(M02,RSG)_RSG | 0.25 |
| Mean Pixel_Valley(M01,BF,3)_BF | 0.25 |
| Area_Threshold(M09,Ch09,30%) | -0.24 |
| H Energy Mean_M02_RSG_1 | 0.23 |
| Bright Detail Intensity R7_MC_PI | 0.23 |
| Symmetry 3_Morphology(M01,BF)_BF | 0.23 |
| Height_Morphology(M01,BF) | 0.23 |
| Intensity_Valley(Object(M01,BF,Tight),BF,3)_BF | 0.23 |
| Height_M01 | 0.23 |
| H Contrast Mean_M01_BF_3 | -0.22 |
| Circularity_Object(M09,Ch09,Tight) | -0.21 |
| Symmetry 3_Morphology(M02,RSG)_RSG | 0.21 |
| H Energy Mean_M01_BF_7 | 0.21 |
| Thickness Min_Object(M09,Ch09,Tight) | -0.21 |
| Intensity_Valley(Morphology(M01,BF),BF,3)_BF | 0.21 |
| Intensity_Valley(M01,BF,3)_BF | 0.21 |
| Symmetry 2_Skeleton(M02,RSG,Thin)_RSG | 0.21 |
| Thickness Max_Object(M09,Ch09,Tight) | -0.21 |
| Symmetry 3_Skeleton(Morphology(M02,RSG),RSG,Thin)_RSG | 0.21 |
| H Homogeneity Mean_M09_Ch09_3 | -0.21 |
| Min Pixel_Valley(M02,RSG,3)_RSG | -0.21 |
| H Contrast Mean_M02_RSG_1 | 0.2 |
| H Entropy Std_M02_RSG_1 | 0.2 |
| Bright Detail Intensity R3_MC_BF | 0.2 |
| Gradient Max_M01_BF | 0.2 |
| H Energy Std_M01_BF_7 | 0.2 |
| Contrast_Object(M01,BF,Tight)_BF | -0.2 |
| Modulation_Object(M09,Ch09,Tight)_Ch09 | -0.19 |
| Diameter_Object(M09,Ch09,Tight) | -0.19 |
| Height_Object(M09,Ch09,Tight) | -0.19 |
| H Correlation Mean_M01_BF_3 | 0.19 |
| Symmetry 3_Object(M01,BF,Tight)_BF | 0.19 |
| H Correlation Std_M01_BF_7 | 0.19 |
| Min Pixel_Valley(M09,Ch09,3)_Ch09 | 0.19 |
| Length_M01 | 0.19 |
| Min Pixel_Valley(Morphology(M09,Ch09),Ch09,3)_Ch09 | 0.19 |
| Lobe Count_Skeleton(Object(M09,Ch09,Tight),Ch09,Thin)_Ch09 | -0.19 |
| Major Axis Intensity_M01_BF | 0.19 |
| Length_Morphology(M01,BF) | 0.19 |
| Elongatedness_Object(M09,Ch09,Tight) | -0.19 |
| Std Dev_M09_Ch09 | -0.18 |
| Major Axis_Morphology(M01,BF) | 0.18 |
| Lobe Count_Object(M09,Ch09,Tight)_Ch09 | -0.18 |
| Circularity_Morphology(M01,BF) | -0.18 |
| Major Axis_M01 | 0.18 |
| Raw Min Pixel_MC_Ch09 | 0.18 |
| Std Dev_Morphology(M09,Ch09)_Ch09 | -0.18 |
| H Correlation Std_M02_RSG_1 | 0.18 |
| Std Dev_Object(M09,Ch09,Tight)_Ch09 | -0.18 |
| Circularity_M01 | -0.18 |
| Lobe Count_Skeleton(Morphology(M09,Ch09),Ch09,Thin)_Ch09 | -0.18 |
| Lobe Count_Skeleton(M09,Ch09,Thin)_Ch09 | -0.18 |
| Min Pixel_Valley(Object(M09,Ch09,Tight),Ch09,3)_Ch09 | 0.18 |
| Symmetry 3_Skeleton(M02,RSG,Thick)_RSG | 0.17 |
| Bright Detail Intensity R7_MC_Ch09 | 0.17 |
| Modulation_Morphology(M09,Ch09)_Ch09 | -0.17 |
| Modulation_M09_Ch09 | -0.17 |
| Mean Pixel_Object(M01,BF,Tight)_BF | 0.17 |
| Area_Object(M09,Ch09,Tight) | -0.17 |
| Mean Pixel_Valley(Object(M01,BF,Tight),BF,3)_BF | 0.17 |
| H Homogeneity Mean_M01_BF_7 | 0.17 |
| Minor Axis Intensity_M01_BF | 0.17 |
| Max Pixel_Skeleton(Object(M01,BF,Tight),BF,Thin)_BF | 0.17 |
| H Contrast Mean_M09_Ch09_3 | -0.16 |
| H Energy Mean_M09_Ch09_3 | -0.16 |
| H Contrast Std_M02_RSG_1 | 0.16 |
| Gradient Max_M09_Ch09 | -0.16 |
| Length_Object(M09,Ch09,Tight) | -0.16 |
| H Homogeneity Std_M01_BF_1 | 0.16 |
| Aspect Ratio_Morphology(M09,Ch09) | -0.16 |
| H Correlation Std_M01_BF_1 | 0.16 |
| Gradient RMS_M09_Ch09 | -0.16 |
| H Contrast Std_M01_BF_3 | -0.16 |
| Perimeter_M01 | 0.16 |
| Aspect Ratio_M09 | -0.16 |
| Min Pixel_Skeleton(Object(M09,Ch09,Tight),Ch09,Thin)_Ch09 | 0.16 |
| Perimeter_Morphology(M01,BF) | 0.16 |
| H Correlation Std_M02_RSG_3 | 0.16 |
| H Entropy Mean_M09_Ch09_3 | -0.16 |
| Max Pixel_Skeleton(Morphology(M01,BF),BF,Thin)_BF | 0.15 |
| Width_Object(M09,Ch09,Tight) | -0.15 |
| Minor Axis_Morphology(M01,BF) | 0.15 |
| H Energy Std_M02_RSG_1 | 0.15 |
| Perimeter_Object(M09,Ch09,Tight) | -0.15 |
| Minor Axis_M01 | 0.15 |
| Mean Pixel_Skeleton(Object(M09,Ch09,Tight),Ch09,Thin)_Ch09 | 0.15 |
| H Variance Std_M02_RSG_1 | 0.15 |
| Min Pixel_Skeleton(Morphology(M09,Ch09),Ch09,Thin)_Ch09 | 0.15 |
| Min Pixel_Skeleton(M09,Ch09,Thin)_Ch09 | 0.15 |
| H Homogeneity Std_M01_BF_7 | 0.15 |
| H Homogeneity Mean_M09_Ch09_1 | -0.15 |
| Minor Axis_Object(M09,Ch09,Tight) | -0.15 |
| Max Pixel_Skeleton(M01,BF,Thin)_BF | 0.15 |
| Elongatedness_M01 | 0.14 |
| Width_M01 | 0.14 |
| Intensity_Skeleton(Object(M09,Ch09,Tight),Ch09,Thin)_Ch09 | 0.14 |
| Aspect Ratio_Object(M09,Ch09,Tight) | -0.14 |
| Elongatedness_Morphology(M01,BF) | 0.14 |
| Circularity_M09 | -0.14 |
| Circularity_Morphology(M09,Ch09) | -0.14 |
| Aspect Ratio Intensity_M09_Ch09 | -0.14 |
| H Contrast Std_M09_Ch09_7 | 0.14 |
| H Contrast Mean_M09_Ch09_7 | 0.14 |
| H Entropy Mean_M01_BF_1 | -0.14 |
| Width_Morphology(M01,BF) | 0.14 |
| Thickness Max_M09 | -0.13 |
| Lobe Count_Skeleton(Object(M01,BF,Tight),BF,Thick)_BF | -0.13 |
| Diameter_M09 | -0.13 |
| H Variance Mean_M09_Ch09_3 | -0.13 |
| Symmetry 3_Skeleton(Object(M09,Ch09,Tight),Ch09,Thick)_Ch09 | -0.13 |
| Compactness_M09_Ch09 | -0.13 |
| H Homogeneity Std_M02_RSG_3 | 0.13 |
| Thickness Max_Morphology(M09,Ch09) | -0.13 |
| H Contrast Std_M09_Ch09_3 | -0.13 |
| H Entropy Std_M01_BF_1 | 0.13 |
| Compactness_Morphology(M09,Ch09)_Ch09 | -0.13 |
| H Entropy Mean_M01_BF_7 | 0.13 |
| Diameter_Morphology(M09,Ch09) | -0.13 |
| H Entropy Mean_M01_BF_3 | -0.13 |
| Lobe Count_Morphology(M09,Ch09)_Ch09 | -0.13 |
| Symmetry 3_Skeleton(Object(M09,Ch09,Tight),Ch09,Thin)_Ch09 | -0.12 |
| Mean Pixel_Object(M09,Ch09,Tight)_Ch09 | 0.12 |
| H Homogeneity Mean_M01_BF_1 | -0.12 |
| H Correlation Std_M09_Ch09_3 | -0.12 |
| Contrast_M09_Ch09 | -0.12 |
| Thickness Max_Object(M01,BF,Tight) | -0.12 |
| Aspect Ratio_Skeleton(Morphology(M09,Ch09),Ch09,Thin) | -0.12 |
| H Variance Mean_M01_BF_1 | -0.12 |
| Symmetry 4_Object(M01,BF,Tight)_BF | 0.12 |
| H Entropy Mean_M09_Ch09_1 | -0.12 |
| Contrast_Morphology(M09,Ch09)_Ch09 | -0.12 |
| Aspect Ratio_Skeleton(M09,Ch09,Thin) | -0.12 |
| Bright Detail Intensity R3_MC_Ch09 | 0.12 |
| Lobe Count_Skeleton(Morphology(M09,Ch09),Ch09,Thick)_Ch09 | -0.11 |
| H Entropy Std_M02_RSG_3 | 0.11 |
| Symmetry 2_Object(M01,BF,Tight)_BF | 0.11 |
| H Contrast Std_M09_Ch09_13 | -0.11 |
| Area_M09 | -0.11 |
| Intensity_Skeleton(M09,Ch09,Thin)_Ch09 | 0.11 |
| H Variance Mean_M09_Ch09_13 | -0.11 |
| Area_Skeleton(Object(M09,Ch09,Tight),Ch09,Thin) | -0.11 |
| H Homogeneity Mean_M01_BF_3 | -0.11 |
| H Variance Std_M09_Ch09_13 | -0.11 |
| H Contrast Mean_M09_Ch09_13 | -0.11 |
| Lobe Count_Skeleton(M09,Ch09,Thick)_Ch09 | -0.11 |
| Thickness Min_Morphology(M09,Ch09) | -0.11 |
| Area_Morphology(M09,Ch09) | -0.11 |
| Thickness Min_M09 | -0.11 |
| Intensity_Skeleton(Morphology(M09,Ch09),Ch09,Thin)_Ch09 | 0.11 |
| Aspect Ratio_Skeleton(Object(M09,Ch09,Tight),Ch09,Thin) | -0.11 |
| Width_M09 | -0.1 |
| Thickness Min_Object(M01,BF,Tight) | -0.1 |
| H Homogeneity Std_M02_RSG_1 | 0.1 |
| H Entropy Std_M09_Ch09_3 | -0.1 |
| Area_Object(M01,BF,Tight) | -0.1 |
| Aspect Ratio Intensity_Skeleton(Object(M09,Ch09,Tight),Ch09,Thin)_Ch09 | -0.1 |
| H Homogeneity Std_M09_Ch09_3 | -0.1 |
| H Energy Std_M09_Ch09_3 | -0.1 |
| H Correlation Mean_M09_Ch09_1 | -0.1 |
| H Correlation Mean_M09_Ch09_3 | -0.1 |
| Aspect Ratio Intensity_M01_BF | -0.1 |
| Compactness_Object(M09,Ch09,Tight)_Ch09 | -0.1 |
| H Correlation Mean_M09_Ch09_7 | -0.1 |
| Symmetry 2_Morphology(M01,BF)_BF | 0.1 |
| Symmetry 2_Skeleton(Morphology(M02,RSG),RSG,Thick)_RSG | 0.1 |
| Symmetry 4_Skeleton(Morphology(M02,RSG),RSG,Thick)_RSG | 0.1 |
| Lobe Count_Skeleton(Morphology(M02,RSG),RSG,Thick)_RSG | 0.1 |
| H Entropy Std_M01_BF_7 | 0.1 |
| Width_Morphology(M09,Ch09) | -0.1 |
| H Variance Std_M09_Ch09_3 | -0.1 |
| Bright Detail Intensity R7_MC_BF | 0.1 |
| Compactness_Object(M01,BF,Tight)_BF | 0.1 |
| Area_Skeleton(Morphology(M09,Ch09),Ch09,Thin) | -0.09 |
| Area_Skeleton(M09,Ch09,Thin) | -0.09 |
| H Correlation Std_M09_Ch09_9 | 0.09 |
| Symmetry 4_Skeleton(Object(M09,Ch09,Tight),Ch09,Thin)_Ch09 | -0.09 |
| Symmetry 2_Skeleton(Object(M09,Ch09,Tight),Ch09,Thin)_Ch09 | -0.09 |
| Thickness Max_M01 | -0.09 |
| Symmetry 4_Skeleton(Object(M01,BF,Tight),BF,Thick)_BF | -0.09 |
| Thickness Min_M01 | -0.09 |
| H Correlation Std_M01_BF_11 | 0.09 |
| Lobe Count_Object(M01,BF,Tight)_BF | 0.09 |
| Thickness Min_Morphology(M01,BF) | -0.09 |
| H Variance Std_M01_BF_7 | 0.09 |
| H Variance Mean_M01_BF_7 | 0.09 |
| H Energy Std_M02_RSG_3 | 0.09 |
| Thickness Max_Morphology(M01,BF) | -0.09 |
| Diameter_Object(M01,BF,Tight) | -0.09 |
| Minor Axis_Morphology(M09,Ch09) | -0.08 |
| Shape Ratio_Morphology(M01,BF) | -0.08 |
| H Contrast Mean_M09_Ch09_17 | 0.08 |
| Shape Ratio_M01 | -0.08 |
| H Entropy Std_M01_BF_3 | -0.08 |
| Symmetry 4_Skeleton(M01,BF,Thin)_BF | 0.08 |
| Symmetry 4_Morphology(M01,BF)_BF | 0.08 |
| Shape Ratio_M09 | -0.08 |
| Symmetry 2_Skeleton(Object(M09,Ch09,Tight),Ch09,Thick)_Ch09 | 0.08 |
| Lobe Count_Skeleton(Morphology(M01,BF),BF,Thick)_BF | -0.08 |
| Symmetry 2_Skeleton(Object(M01,BF,Tight),BF,Thick)_BF | -0.08 |
| Median Pixel_M01_BF | 0.08 |
| Mean Pixel_Skeleton(Morphology(M09,Ch09),Ch09,Thin)_Ch09 | 0.08 |
| Aspect Ratio Intensity_Skeleton(Morphology(M09,Ch09),Ch09,Thin)_Ch09 | -0.08 |
| H Variance Std_M09_Ch09_17 | 0.08 |
| Lobe Count_Skeleton(M01,BF,Thick)_BF | -0.08 |
| Mean Pixel_Valley(Object(M09,Ch09,Tight),Ch09,3)_Ch09 | 0.08 |
| Max Pixel_Valley(Morphology(M09,Ch09),Ch09,3)_Ch09 | -0.08 |
| H Variance Mean_M01_BF_9 | 0.08 |
| H Contrast Std_M09_Ch09_17 | 0.08 |
| Mean Pixel_Skeleton(M09,Ch09,Thin)_Ch09 | 0.08 |
| Max Pixel_Valley(Object(M09,Ch09,Tight),Ch09,3)_Ch09 | -0.08 |
| Aspect Ratio Intensity_Skeleton(M09,Ch09,Thin)_Ch09 | -0.08 |
| H Variance Std_M01_BF_9 | 0.08 |
| Circularity_Object(M01,BF,Tight) | -0.08 |
| H Energy Std_M09_Ch09_1 | -0.08 |
| Shape Ratio_Morphology(M09,Ch09) | -0.08 |
| Median Pixel_M09_Ch09 | -0.08 |
| H Correlation Mean_M09_Ch09_9 | -0.08 |
| Max Pixel_Valley(M09,Ch09,3)_Ch09 | -0.08 |
| H Correlation Std_M09_Ch09_7 | 0.08 |
| Minor Axis_M09 | -0.08 |
| Symmetry 4_Skeleton(Morphology(M01,BF),BF,Thin)_BF | 0.08 |
| H Variance Mean_M09_Ch09_17 | 0.08 |
| Max Pixel_Skeleton(Object(M09,Ch09,Tight),Ch09,Thin)_Ch09 | 0.08 |
| H Correlation Mean_M01_BF_11 | -0.07 |
| Symmetry 3_Object(M09,Ch09,Tight)_Ch09 | -0.07 |
| H Homogeneity Std_M01_BF_11 | 0.07 |
| H Contrast Std_M01_BF_9 | 0.07 |
| Symmetry 2_Skeleton(Morphology(M01,BF),BF,Thin)_BF | 0.07 |
| H Correlation Std_M01_BF_5 | 0.07 |
| Symmetry 2_Skeleton(M01,BF,Thin)_BF | 0.07 |
| Aspect Ratio_Object(M01,BF,Tight) | -0.07 |
| H Variance Std_M01_BF_5 | 0.07 |
| H Contrast Mean_M01_BF_7 | 0.07 |
| Intensity_Valley(Object(M09,Ch09,Tight),Ch09,3)_Ch09 | 0.07 |
| H Entropy Mean_M09_Ch09_7 | 0.07 |
| H Homogeneity Mean_M09_Ch09_5 | -0.07 |
| H Homogeneity Mean_M09_Ch09_13 | -0.07 |
| H Homogeneity Std_M09_Ch09_5 | -0.07 |
| H Homogeneity Std_M09_Ch09_13 | -0.07 |
| H Energy Mean_M09_Ch09_7 | 0.07 |
| Perimeter_M09 | -0.07 |
| H Variance Mean_M09_Ch09_1 | -0.07 |
| Aspect Ratio_M01 | -0.07 |
| H Contrast Mean_M01_BF_9 | 0.07 |
| Aspect Ratio_Morphology(M01,BF) | -0.07 |
| Raw Min Pixel_MC_RSG | -0.07 |
| Symmetry 4_Skeleton(M09,Ch09,Thin)_Ch09 | -0.07 |
| Perimeter_Morphology(M09,Ch09) | -0.07 |
| Symmetry 4_Skeleton(Morphology(M09,Ch09),Ch09,Thin)_Ch09 | -0.07 |
| H Energy Std_M01_BF_5 | 0.07 |
| Symmetry 2_Skeleton(M02,RSG,Thick)_RSG | 0.07 |
| Max Pixel_Valley(Morphology(M01,BF),BF,3)_BF | -0.06 |
| Symmetry 2_Skeleton(Morphology(M01,BF),BF,Thick)_BF | -0.06 |
| Symmetry 3_Skeleton(Object(M01,BF,Tight),BF,Thin)_BF | 0.06 |
| Max Pixel_Valley(M01,BF,3)_BF | -0.06 |
| Symmetry 4_Skeleton(M01,BF,Thick)_BF | -0.06 |
| Symmetry 3_Skeleton(Morphology(M01,BF),BF,Thick)_BF | 0.06 |
| Symmetry 3_Skeleton(M01,BF,Thick)_BF | 0.06 |
| Symmetry 4_Skeleton(Morphology(M01,BF),BF,Thick)_BF | -0.06 |
| H Contrast Std_M01_BF_5 | 0.06 |
| Symmetry 2_Skeleton(M01,BF,Thick)_BF | -0.06 |
| H Variance Mean_M01_BF_13 | 0.06 |
| H Variance Std_M01_BF_13 | 0.06 |
| H Energy Std_M09_Ch09_7 | 0.06 |
| H Energy Mean_M01_BF_5 | 0.06 |
| H Variance Std_M01_BF_1 | -0.06 |
| H Contrast Mean_M01_BF_5 | 0.06 |
| H Contrast Mean_M01_BF_13 | 0.06 |
| Elongatedness_Object(M01,BF,Tight) | 0.06 |
| H Correlation Mean_M09_Ch09_11 | 0.06 |
| H Contrast Std_M01_BF_7 | 0.06 |
| H Homogeneity Mean_M01_BF_11 | 0.06 |
| Contrast_Object(M09,Ch09,Tight)_Ch09 | -0.06 |
| H Correlation Mean_M01_BF_5 | -0.06 |
| H Energy Std_M09_Ch09_9 | 0.06 |
| H Entropy Mean_M09_Ch09_13 | -0.06 |
| H Variance Mean_M01_BF_5 | 0.06 |
| H Entropy Std_M09_Ch09_13 | -0.06 |
| H Contrast Std_M01_BF_13 | 0.06 |
| Min Pixel_Valley(Morphology(M02,RSG),RSG,3)_RSG | 0.06 |
| H Homogeneity Std_M09_Ch09_1 | -0.05 |
| H Contrast Std_M09_Ch09_11 | -0.05 |
| H Energy Mean_M01_BF_11 | 0.05 |
| Symmetry 4_Skeleton(M02,RSG,Thick)_RSG | 0.05 |
| Minor Axis_Object(M01,BF,Tight) | -0.05 |
| Length_Morphology(M09,Ch09) | -0.05 |
| Area_Threshold(M01,BF,30%) | -0.05 |
| H Correlation Std_M01_BF_9 | 0.05 |
| Shape Ratio_Object(M09,Ch09,Tight) | -0.05 |
| Width_Object(M01,BF,Tight) | -0.05 |
| Length_M09 | -0.05 |
| H Contrast Mean_M09_Ch09_11 | -0.05 |

Table S3. DEGs classified into the ‘hypothetical proteins’ group of *P. aeruginosa* NT06 exposed to KCl (A) and KCl/NaL/NaC (D) under microaerophilic conditions

| Name | Description | Log2-FC | |
| --- | --- | --- | --- |
|  |  | A | D |
| PA4726.2 | hypothetical protein p30, small ncRNA | 11.02 | – |
| PA0819 | hypothetical protein | 0.82 | 4.32 |
| PA1414 | hypothetical protein | 0.82 | 3.94 |
| PA0111 | probable transmembrane protein | 3.08 | – |
| PA0952 | probable transcriptional regulator | 2.95 | – |
| PA0109 | probable twin transmembrane helix small protein | 2.64 | – |
| PA0713 | DUF2845 domain-containing protein | 2.60 | 3.09 |
| PA4141 | hypothetical protein | – | 2.60 |
| PA2805 | probable DUF4404 family protein | 2.58 | 2.44 |
| PA4139 | hypothetical protein | – | 2.51 |
| PA0365 | hypothetical protein | 2.48 | – |
| PA1870 | hypothetical protein | 2.48 | – |
| PA3273 | probable transcriptional regulator protein | 2.45 | 2.22 |
| PA3371 | DUF4175 domain-containing protein | 2.45 | – |
| PA1123 | DUF2025 family protein | – | 2.23 |
| PA1852 | DUF1508 domain-containing protein | 2.17 | 1.74 |
| PA0736 | DUF2157 domain-containing protein | 2.15 | 1.90 |
| PA4607 | DUF3015 domain-containing protein | 2.08 | 1.89 |
| PA0200 | DUF3079 domain-containing protein | 1.43 | 1.95 |
| PA0050 | probable secreted protein | 1.93 | 2.88 |
| PA3369 | probable secreted protein | 1.92 | – |
| PA1414 | uncharacterized protein | 1.91 | 3.94 |
| PA1076 | DUF5064 domain-containing protein | 1.30 | 1.84 |
| PA3051 | DUF2835 domain-containing protein | – | 1.84 |
| PA4793 | DUF4136 domain-containing protein | – | 1.84 |
| PA2781 | hypothetical protein | 1.80 | 1.73 |
| PA5475 | GNAT family N-acetyltransferase | 1.19 | 1.78 |
| PA1075 | DUF2288 family protein | – | 1.74 |
| PA1749 | GNAT family N-acetyltransferase | 1.18 | 1.74 |
| PA0714 | hypothetical protein | – | 1.70 |
| PA5226 | TIGR02449 family protein with unknown function | – | 1.67 |
| PA2747 | DUF3509 domain-containing protein | 1.64 | – |
| PA0329 | YegP family protein with uncharacterized function | – | 1.61 |
| PA4573 | DUF6482 family protein | 1.61 | – |
| PA3235 | DUF485 domain-containing protein | 1.60 | – |
| PA0981 | hypothetical protein | – | 1.57 |
| PA4775 | hypothetical protein | – | 1.57 |
| PA2504 | DUF2185 domain-containing protein, partial | 1.56 | – |
| PA0543 | probable polyhydroxybutyrate depolymerase | 1.51 | – |
| PA0769 | DUF4845 domain-containing protein | 1.28 | 1.51 |
| PA2422 | DUF3077 domain-containing hypothetical protein | – | -1.62 |
| PA5104 | HutD family protein with unknown function | -1.66 | -2.33 |
| PA4940 | DUF2065 domain-containing protein | -1.67 | – |
| PA4387 | Membrane protein FxsA | -1.71 | – |
| PA1345 | RimK family alpha-L-glutamate ligase | -1.7 | – |
| PA3730 | hypothetical proteinr | – | -1.76 |
| PA1450 | YgcG family protein | – | -1.76 |
| PA1652 | hypothetical protein, DUF2157 domain-containing protein | -1.7 | – |
| PA1018 | MaoC/PaaZ C-terminal domain-containing protein | – | -1.85 |
| PA3518 | HOASN domain-containing protein | -2.1 | – |
| PA5031 | Probably oxidoreductase with unknown function | – | -3.19 |

Predicted functions based on sequence homology were searched among available gene and protein databases. Red color indicates values with log2-FC < |1.5|. ‘–‘ indicates not present

Table S4. DEGs classified to ‘tRNA and rRNA’ group of *P. aeruginosa* NT06 exposed to KCl (A) and KCl/NaL/NaC (D)

| Name | Gene product and function | Log2-FC | |
| --- | --- | --- | --- |
|  |  | A | D |
| PA2819.2 | tRNA-Gly | 4.15 | 4.60 |
| PA0976.1 | tRNA-Lys | 3.45 | 5.41 |
| PA3031.1 | tRNA-Pro | 3.03 | 4.94 |
| PA5160.1 | tRNA-Tyr | – | 3.85 |
| PA4802.1 | tRNA-Sec | – | 3.47 |
| PA4669.1 | tRNA-Gln | – | 3.32 |
| ssrS | 6S RNA | 2.05 | 3.13 |
| PA2736.1 | tRNA-Pro | 2.04 | 3.25 |
| ffs | 4.5S RNA | 1.89 | 3.12 |
| PA4937.2 | tRNA-Leu | 1.60 | 3.09 |
| PA3824.1 | tRNA-Leu | – | 2.85 |
| PA2603.1 | tRNA-Ser | – | 2.80 |
| PA4937.2 | tRNA-Leu | – | 2.80 |
| PA1796.3 | tRNA-Leu | – | 2.70 |
| PA4746.2 | tRNA-Leu | – | 2.63 |
| PA3262.2 | tRNA-Leu | – | 2.62 |
| PA4276.1 | tRNA-Trp | – | 2.51 |
| PA3094.3 | tRNA-Val | – | 2.24 |
| PA0922.1 | tRNA-Met | – | 2.01 |
| PA0668.3 | tRNA-Ala | – | 1.81 |
| PA3139.1 | tRNA-Asn | – | 1.78 |
| PA2581.1 | tRNA-Cys | – | 1.74 |
| PA0729.1 | tRNA-Gly | – | 1.72 |
| PA5149.1 | tRNA-Phe | – | 1.66 |
| PA4277.3 | tRNA-Tyr | – | 1.62 |
| PA4280.2 | 23S ribosomal RNA | -1.60 | – |
| PA3133.2 | tRNA-Ala | -1.63 | – |
| rimI | ribosomal protein S18-alanine N-acetyltransferase | -1.78 | 2.27 |
| PA0668.4 | 23S ribosomal RNA | – | -2.20 |
| PA4280.2 | 23S ribosomal RNA | – | -2.25 |
| PA4673.1 | tRNA-Met | -2.65 | – |
| PA3133.4 | tRNA-Ala | -3.04 | – |

‘–‘ indicates not present

Table S5. DEGs classified into the ‘cell maintenance and division’ group of *P. aeruginosa* NT06 exposed to KCl (A) and KCl/NaL/NaC (D) under microaerophilic conditions

| Name | Gene product and function | Log2-FC | |
| --- | --- | --- | --- |
|  |  | A | D |
| PA3431 | LrgB family protein involved in murein hydrolase activity | – | 3.66 |
| PA3432 | CidA/LrgA family protein involved in cell death and lysis | – | 3.28 |
| rnpB | RNA component of endoribonuclease RNaseP involved in removal of the 5′-leader of precursor tRNAs | 2.38 | 1.79 |
| rmf | ribosome modulation factor, involved in negative regulation of translation | 1.99 | 2.84 |
| PA4463 | ribosome-associated translation inhibitor RaiA involved in negative regulation of translational elongation | 1.71 | – |
| PA4441 | YhcB family protein, a membrane protein involved in cell morphology homeostasis and cell division | – | 1.68 |
| efp | translation elongation factor P, upregulated at low temperatures | 1.66 | – |
| PA3940 | HU family DNA-binding protein, structural constituent of chromatin | 1.57 | 1.67 |
| xerD | tyrosine recombinase XerD, involved in conversion of dimers of the bacterial chromosome into monomers to permit their segregation at cell division | -1.67 | – |
| PA3559 | UDP-glucose 6-dehydrogenase | – | 1.64 |
| PA5028 | probable parA, chromosome segregation ATPase | – | 1.55 |
| PA5228 | 5-formyltetrahydrofolate cyclo-ligase | – | -1.61 |
| codB | cytosine permease involved in transport of cytosine, that build the DNA and RNA | -1.70 | -1.66 |
| PA0017 | 16S rRNA (cytosine(967)-C(5))-methyltransferase RsmB | – | -1.74 |
| PA5456 | glycosyltransferase involved in cell wall biogenesis | -1.80 | – |
| PA3517 | purine biosynthesis pathway for de novo synthesis of IMP | – | -1.83 |
| PA0670 | DNA polymerase Y family protein, involved in DNA repair and SOS response | – | -1.86 |
| PA1135 | protein deglycase HchA | – | -1.91 |
| PA3596 | trifunctional transcriptional activator/DNA repair protein Ada/methylated-DNA--[protein]-cysteine S-methyltransferase | – | -2.50 |
| PA5470 | peptide chain release factor H | – | -2.60 |

Predicted functions based on sequence homology were searched among available gene and protein databases. ‘–‘ indicates not present

Table S6. DEGs classified into the ‘stress response and Fe, Cu homeostasis’ group of *P. aeruginosa* NT06 exposed to KCl (A) and KCl/NaL/NaC (D) under microaerophilic conditions

| Name | Gene product and function | Log2-FC | |
| --- | --- | --- | --- |
|  |  | A | D |
| rubA1 | rubredoxin 1, part of RdxR–Rdx system, part of the oxidative stress response | 2.23 | – |
| PA4359 | ferrous iron transporter A | – | 2.66 |
| PA4357 | FeoC domain-containing protein involved in iron acquisition from environment | 2.06 | 2.49 |
| PA3530 | bacterioferritin-associated ferredoxin Bfd | – | 2.40 |
| PA1190 | Yip1 domain-containing protein, involved in response to oxidative stress | 2.00 | – |
| PA4925 | mechanosensitive ion channel family protein that contribute to resistance to osmotic stress | 1.93 | – |
| PA2827 | peptide-methionine (R)-S-oxide reductase MsrB, involved in response to oxidative stress | 1.85 | 2.36 |
| PA4739 | BON domain-containing protein involved in response to osmotic stress, antibiotic resistance and virulence | 1.77 | 1.26 |
| ccpR | cytochrome c551 peroxidase precursor, oxidoreductase activity, provide protection against toxic peroxides | 1.65 | 2.81 |
| sodM | superoxide dismutase involved in superoxide radicals degradation | – | 2.05 |
| PA5217 | putative binding protein of iron ABC transporter | 1.52 | 1.69 |
| PA2864 | DoxX family protein involved in radical detoxification | – | 1.75 |
| PA4352 | universal stress protein, essential for survival during anaerobic and stress conditions | 0.82 | 1.68 |
| PA0102 | beta-carbonic anhydrase involved in adaptation to CO_2_ conditions | – | 1.65 |
| trxB2 | Thioredoxin reductase involved in cell redox homeostasis by removal of superoxide radicals | 1.50 | 1.60 |
| PA1789 | UspA domain-containing protein, putative universal stress protein, involved in survival during anaerobic growth | 1.00 | 1.57 |
| PA1297 | CDF family iron/cobalt efflux transporter AitP | -1.64 | – |
| PA3888 | OpuC ABC transporter, permease protein, OpuCD | – | -1.76 |
| PA1134 | thiol-disulfide oxidoreductase DCC family protein, crucial for cell redox homeostasis | -1.80 | – |
| betT1 | BCCT family transporter BetT1, involved in uptake of choline under hyperosmolar conditions | – | -1.93 |
| PA3574a | copper chaperone CopZ involved in copper homeostasis | – | -2.16 |
| PA3920 | probable copper-translocating P-type ATPase CopA1, involved in copper homeostasis | -2.29 | -2.65 |

Predicted functions based on sequence homology were searched among available gene and protein databases. Red color indicates values with log2-FC < |1.5|. ‘–‘ indicates not present

Table S7. DEGs classified into the ‘biofilm, quorum sensing and virulence’ group of *P. aeruginosa* NT06 exposed to KCl (A) and KCl/NaL/NaC (D) under microaerophilic conditions

| Name | Gene product and function | Log2-FC | |
| --- | --- | --- | --- |
|  |  | A | D |
| PA1869 | acp2, probably acyl carrier protein involved in fatty acid biosynthetic process and quorum sensing signals | 3.01 | 2.99 |
| PA0122 | rahU - aegerolysin family protein, binds with glycolypid (rhamnolipid), in host induce hemolysis of red blood cells | 2.80 | 2.04 |
| pilA | type IV fimbrial precursor PilA | – | 2.51 |
| pilC | inner membrane component of the type IV pilus system, controlling both pilus assembly and disassembly | 1.23 | 2.23 |
| PA1041 | OmpA family protein, involved in bacterial adhesion, invasion, or intracellular survival | 2.34 | 1.82 |
| PA2780 | bacterial swarming regulator BswR involved in negative regulation of cell motility and biofilm | 2.25 | 1.97 |
| PA1784 | polysaccharide lyase family 7 protein, alginate lyase2 involved in alginate degradation | 2.23 | – |
| oprI | Major outer membrane lipoprotein OprI, that induce cytokine production | 2.18 | 2.18 |
| osmE | osmotically inducible lipoprotein OsmE having proinflammatory potential | 2.04 | 1.04 |
| PA1169 | arachidonate 15-lipoxygenase, converts arachidonic acid into 15-hydroxyeicosatetraenoic acid, modulate host defense and inflammation | – | 2.16 |
| PA3908 | type VI secretion system immunity protein, TsiT, antitoxin for TseT, which is toxic for other cells | 1.99 | – |
| PA0045 | CsgG - lipoprotein that forms secretion channel for curli, "amyloids" that are important in biofilm formation, host cell adhesion and colonization | 1.97 | – |
| hemE | uroporphyrinogen decarboxylase, involved in heme biosynthesis that is required in pathogenesis | 1.69 | – |
| fimU | type IV fimbrial biogenesis protein FimU involved in type IV pilus-dependent motility | 1.69 | 2.22 |
| bdlA | Biofilm dispersion protein BdlA, involved the modulation of c-di-GMP levels, swimming motility and adhesiveness of the bacterial cell to surface | 1.60 | – |
| PA0626 | phage tail protein | 1.57 | – |
| tadZ | type IV pilus Flp biogenesis protein TadZ | 1.57 | – |
| lasB | elastase LasB involved in quorum sensing-dependent proteolysis | 1.54 | 0.93 |
| rhlA | rhamnosyltransferase chain A involved in rhamnolipid biosynthesis | 1.53 | 2.37 |
| aprI | alkaline proteinase inhibitor AprI, involved in negative regulation of endopeptidase activity | 1.53 | 1.45 |
| PA4833 | hemolysin III family protein | – | 1.96 |
| PA3919 | PhoH family protein, PhoH is a cytoplasmic protein and predicted ATPase that is induced by phosphate starvation | 1.38 | 1.90 |
| PA5526 | YgdI/YgdR family lipoprotein | – | 1.87 |
| FlgB | Flagellar basal body rod protein FlgB | – | 1.83 |
| PA1245 | OmpA family protein, involved in bacterial adhesion, invasion, or intracellular survival | – | 1.82 |
| flgF | Flagellar basal body rod protein FlgF | – | 1.72 |
| oprC | outer membrane porin OprC precursor, siderophore transmembrane transport | 1.11 | 1.67 |
| PA3559 | UDP-glucose 6-dehydrogenase involved in the synthesis of exopolysaccharides (EPS) and lipopolysaccharides (LPS) critical for bacterial virulence | 1.09 | 1.64 |
| tonB1 | TonB protein involved in iron import into cell, protein and siderophore transport, biofilm formation and type IV-pilus dependent-motility | – | 1.61 |
| PA0572 | ImpA family metalloprotease involved in degradation of the host immune system proteins | – | 1.61 |
| PA0084 | TssC1 protein involved in type VI secretion system and biofilm-related antibiotic resistance | 1.03 | 1.61 |
| PA1658 | Type VI secretion system contractile sheath large subunit | 1.26 | 1.53 |
| PA4108 | cyclic di-GMP phosphodiesterase | – | 1.50 |
| PA1952 | acessory protein FapE, a part of the functional amyloid system (Fap) involved in stabilization of biofilm | -1.56 | -2.63 |
| PA2558 | MgtC/SapB family protein probably virulence factor required for growth in low Mg2+ medium | -1.62 | – |
| PA0757 | Sensor protein QseC, involved in activation in virulence genes in response to interkingdom signal compounds | -1.62 | -1.13 |
| PA1181 | Diguanylate cyclase which participate in formation of the cyclic-di-GMP involved in biofilm formation and persistence | -1.70 | -1.53 |
| PA1356 | exo-alpha-sialidase, involved in pathogenesis by providing nutrients and promoting colonization, adhesion, and biofilm formation | -1.30 | -1.58 |
| PA3242 | lipid A biosynthesis lauroyl acyltransferase | -1.26 | -1.68 |
| PA3239 | VacJ family lipoprotein | – | -1.92 |
| PA1209 | DedA family protein/thiosulfate sulfurtransferase GlpE involved in transfer of sulphur, downregulation contribute to decreased virulence | – | -2.04 |
| PA3360 | HlyD family secretion protein involved in the transport of hemolysin | – | -2.10 |
| PA2774 | Tse4, toxic effector protein secreted by the type VI secretion system | -2.07 | – |
| PA0080 | Type VI secretion system lipoprotein TssJ | -2.09 | -2.77 |
| PA1021 | Probable enoyl-CoA hydratase/isomerase involved in fatty acid degradation | -1.73 | -4.57 |

Predicted functions based on sequence homology were searched among available gene and protein databases. Red color indicates values with log2 FC < |1.5|. ‘–‘ indicates not present

Table S8. DEGs encoding ‘transcriptional regulators’ group of *P. aeruginosa* NT06 exposed to KCl (A) and KCl/NaL/NaC (D) under microaerophilic conditions

| Name | Gene product and function | Log2-FC | |
| --- | --- | --- | --- |
|  |  | A | D |
| prrF2 | regulatory RNA PrrF2 involved in iron homeostasis, promote the production of 2-akyl-4(1H)-quinolone metabolites that mediate quorum sensing | – | 3.14 |
| rsmY | small ncRNA class, regulator of GacS/GacA system, part of quorum sensing | 2.76 | 2.56 |
| pprB | two-component response regulator PprB, positive regulation of cell adhesion and biofilm | 2.59 | 1.69 |
| pfeR | two-component response regulator PfeR which activates expression of the ferric enterobactin receptor (siderophore) | – | 2.39 |
| PA4987 | cupin-domain containing protein; xenobiotic response element involved in transcriptional regulation | 2.30 | – |
| liuR | liu genes transcriptional regulator LiuR involved in isoprenoid catabolic process | – | 2.11 |
| rgsA | RgsA small ncRNA, involved in oxidative stress resistance and negative regulation of bacterial-type flagellum-dependent cell motility | 2.06 | 1.70 |
| crcZ | CrcZ, small ncRNA, increased in anaerobic parts of biofilm | 1.99 | 1.77 |
| PA2072 | Bifunctional diguanylate cyclase/phosphodiesterase having signal transduction role in regulating cellular processes | 1.76 | 1.23 |
| rsaL | Regulatory protein RsaL, repressor of quorum sensing and virulence | 1.70 | 2.09 |
| PA3326 | ATP-dependent Clp protease proteolytic subunit 2, related with regulation of virulence genes | 1.51 | – |
| PA2667 | MvaU involved in negative regulation of secondary metabolite biosynthetic process, e.g. pyocyanin synthesis | 1.48 | 2.00 |
| mvaT | transcriptional regulator MvaT, P16 subunit, that negatively regulates biofilm, antimicrobial resistance and fimbrial synthesis | 1.67 | 1.80 |
| phrS | small ncRNA PhrS, a regulatory link between oxygen availability and quorum sensing; impact on oxygen-limited growth in *P. aeruginosa* biofilms. | 1.58 | 1.79 |
| fis | DNA-binding transcriptional regulator Fis, which play a part in the regulation of virulence factors | – | 1.84 |
| PA4611 | YdcH family protein | 0.98 | 1.73 |
| PA3458 | MarR-type transcriptional regulator, involved in osmoadaptation of cells | 1.35 | 1.73 |
| PA4290 | methyl-accepting chemotaxis protein, involved in signal transduction | 1.58 | 1.48 |
| PA2384 | probable transcriptional repressor | – | 1.67 |
| PA4608 | mapZ protein, cyclic-di-GMP binding | 1.53 | 1.56 |
| PA4341 | probable IclR family transcriptional regulator, regulation of multidrug resistance, degradation of aromatics, inactivation of quorum-sensing signals | 1.53 | – |
| trpI | trpBA operon transcriptional activator TrpI, involved in tryptophan synthesis | – | -1.58 |
| PA0528 | LysR substrate-binding domain-containing protein, a probable transcriptional regulator | – | -1.58 |
| mmsR | MmsAB operon regulatory protein, involved in positive regulation of amino acid biosynthetic process | -1.57 | -2.11 |
| PA4989 | LysR family transcriptional regulator | -1.62 | – |
| PA0272 | probable transcriptional regulator | -1.73 | – |
| pcaQ | transcriptional regulator PcaQ, LysR transcriptional regulatory family, regulate the expression of genes involved in aromatic compounds degradation | -1.75 | -2.34 |
| PA3220 | probable transcriptional regulator | -1.83 | – |
| PA0797 | GntR family transcriptional regulator | -2.12 | -1.04 |
| PA1351 | RNA polymerase sigma factor | -2.16 | -1.28 |
| PA1347 | LuxR C-terminal-related transcriptional regulator, involved in quorum sensing and virulence | -2.39 | -1.58 |
| PA1182 | probable transcriptional regulator | -2.89 | -1.16 |
| PA1022 | helix-turn-helix transcriptional regulator | – | -2.94 |
| PA1229 | helix-turn-helix transcriptional regulator | – | -3.03 |

Predicted functions based on sequence homology were searched among available gene and protein databases. Red color indicates values with log2 FC < |1.5|. ‘–‘ indicates not present

Table S9. DEGs encoding ‘antimicrobial resistance and efflux pumps’ of *P. aeruginosa* NT06 exposed to KCl (A) and KCl/NaL/NaC (D) under microaerophilic conditions

| Name | Gene product and function | Log2-FC | |
| --- | --- | --- | --- |
|  |  | A | D |
| PA4313a | transmembrane domain-containing protein, involved in the synthesis of lysyl-phosphatidylglycerol that contribute to resistance to CAP and bacteriocins | – | 2.98 |
| PA1559 | cationic peptide resistance protein CprA | 1.60 | 1.75 |
| PA1170 | TerC family protein involved in resistance to tellurium | – | 1.60 |
| PA1797 | serine hydrolase domain-containing protein, probable beta-lactamase-related domain-containing protein involved in antimicrobial resistance | -1.69 | -1.59 |
| PA1797a | serine hydrolase domain-containing protein, probable beta-lactamase-related domain-containing protein involved in antimicrobial resistance | -1.52 | – |
| PA3523 | multidrug efflux RND transporter periplasmic adaptor subunit MexP, membrane fusion protein of the MexPQ-OpmE | -1.57 | -3.17 |
| PA3522 | multidrug efflux RND transporter permease subunit MexQ, inner membrane transporter of the multidrug efflux pump MexPQ-OpmE | -1.66 | -2.05 |
| PA3521 | multidrug efflux transporter outer membrane subunit OpmE, part of the multidrug efflux pump MexPQ-OpmE | -1.66 | -2.78 |
| oprN | multidrug efflux RND transporter outer membrane subunit OprN, the outer membrane channel component of the MexEF-OprN multidrug efflux complex | – | -1.72 |
| armR | antirepressor for MexR, ArmR, regulating the function of MexAB-OprM efflux pump | -1.83 | -1.42 |
| opmQ | pyoverdine export/recycling transporter outer membrane subunit OmpQ | -1.93 | – |
| PA2837 | efflux transporter outer membrane subunit | – | -1.92 |
| oprJ | probable multidrug efflux transporter outer membrane subunit OprJ, the component of the MexCD-OprJ multidrug efflux complex | – | -1.93 |
| PA2479 | Dsb-associated response regulator, DsbR involved in the cooper resistance | -1.38 | -2.03 |
| nalD | efflux system transcriptional repressor NalD, repressor of MexAB-OprM, involved in negative regulation of transport | -1.22 | -2.36 |

Predicted functions based on sequence homology were searched among available gene and protein databases. Red color indicates values with log2 FC < |1.5|. ‘–‘ indicates not present

Table S10. DEGs involved in ‘transport' of *P. aeruginosa* NT06 exposed to KCl (A) and KCl/NaL/NaC (D) under microaerophilic conditions

| Name | Gene product and function | Log2-FC | |
| --- | --- | --- | --- |
|  |  | A | D |
| PA3213 | MlaD family protein, ABC-type transporter Mla maintaining outer membrane lipid asymmetry | – | 2.22 |
| PA5097 | probable amino acid permease, involved in transport of amino acid into the cell | 1.70 | – |
| PA2982 | biopolymer transporter ExbD required for energy supply and involved in transport of eg. siderophores | 1.67 | – |
| opdO | pyroglutamate porin OpdO | 1.62 | – |
| PA3749 | probable MFS transporter | -1.27 | -1.51 |
| rbsA | Ribose transport protein RbsA, blongs to sugar ABC transporter ATP-binding protein involved in ATP hydrolysis | -1.15 | -1.51 |
| PA1541 | multidrug/spermidine efflux SMR transporter subunit MdtJ | -2.29 | -1.53 |
| PA3670 | GldG family protein, Gliding-associated putative ABC transporter substrate-binding, probably involved in virulence | – | -1.63 |
| PA1519 | solute carrier family 23 protein involved in transport of small molecules | -1.61 | – |
| dctA | C4-dicarboxylate transport protein 2; transport of dicarboxylates (succinate, fumarate, malate) from the periplasm across the membrane | -1.70 | – |
| PA3474 | probable carboxylate/amino acid/amine transporter | -1.72 | – |
| PA3779 | TRAP transporter substrate-binding protein DctP | – | -1.76 |
| PA4504 | dipeptide ABC transporter permease DppC | -1.80 |  |
| PA2563 | SulP family inorganic anion transporter | – | -2.19 |
| PA3937 | probable ATP-binding component of ABC taurine transporter | -2.20 | – |
| PA4622 | probable MFS transporter | – | -2.24 |
| PA4046 | hypothetical protein, MFS transporter | – | -2.36 |
| PA2836 | probable secretion protein | -2.81 | – |
| PA1352 | MFS transporter | -1.91 | -2.86 |

Predicted functions based on sequence homology were searched among available gene and protein databases. Red color indicate values with log2-FC < |1.5|. ‘–‘ indicate not present

Table S11. DEGs involved in ‘amino acid, protein, carbon and lipid metabolism' of *P. aeruginosa* NT06 exposed to KCl (A) and KCl/NaL/NaC (D) under microaerophilic conditions

| Name | Gene product and function | Log2-FC | |
| --- | --- | --- | --- |
|  |  | A | D |
| PA2108 | thiamine pyrophosphate-requiring protein, probably decarboxylase involved in carboxylic acid metabolic process | 1.89 | – |
| PA4774 | Polyamine aminopropyltransferase 2, which catalyzes the spermidine synthesis from putrescine | 1.39 | 1.84 |
| PA3430 | class II aldolase/adducin family protein involved in monosaccharide metabolic process | – | 1.75 |
| arcB | ornithine carbamoyltransferase, catabolic, induced during anaerobic conditions, involved in amino acid degradation | 0.97 | 1.73 |
| arcC | carbamate kinase involved in degradation of arginine at anaerobic conditions with formation of ammonia | 1.14 | 1.67 |
| lldD | L-lactate dehydrogenase | – | 1.64 |
| hutU | urocanate hydratase, involved in amino acid degradation, upregulated at low temperatures | 1.70 | 1.04 |
| hisB | imidazoleglycerol-phosphate dehydratase, involved in histidine biosynthesis | 1.61 | – |
| PA0747 | CoA-acylating methylmalonate-semialdehyde dehydrogenase involved in valine, leucine and isoleucine degradation | 1.43 | 1.58 |
| leuB | 3-isopropylmalate dehydrogenase | – | 1.57 |
| leuD | 3-isopropylmalate dehydratase small subunit | – | 1.56 |
| PA5185 | thioester hydrolase family protein | – | 1.52 |
| PA1830 | probable SCP2 sterol-binding domain-containing protein | – | 1.51 |
| PA2530 | Metalloprotease TldD/E C-terminal domain-containing protein | – | -1.53 |
| PA0142 | 8-oxoguanine deaminase involved in hydrolysis, acting on C-N bond, products are N-isopropylammelide and ethylamine (ammonia like odour) | -1.53 | -1.78 |
| PA2682 | dienelactone hydrolase family protein involved in degradation of chlorocatechol | – | -1.54 |
| PA0924 | sulfatase-like hydrolase/transferase involved in hydrolysis of a sulfuric ester bond | -1.04 | -1.57 |
| PA3586 | alpha/beta hydrolase probably having a triglyceride lipase activity | -1.61 | – |
| pgl | 6-phosphogluconolactonase, involved in carbohydrate degradation during pentose phosphate pathway | -1.33 | -1.63 |
| ilvA2 | threonine dehydratase that catalyzes the formation of alpha-ketobutyrate and ammonia from threonine | -1.65 | -1.43 |
| glcD | glycolate oxidase subunit GlcD | 1.48 | -1.68 |
| glcE | glycolate oxidase subunit GlcE | – | -2.16 |
| PA3226 | alpha/beta hydrolase | -1.70 | -1.32 |
| PA1566 | protein PauA3, involved in catabolism of polyamines | – | -1.77 |
| pauA | acetate--CoA ligase family protein, involved in catabolism of polyamines, like PUT and CAD | -1.02 | -1.80 |
| PA1628 | probable 3-hydroxyacyl-CoA dehydrogenase | -1.99 | – |
| PA3516 | adenylosuccinate lyase family protein, probably involved in carbon metabolism under anaerobic conditions | -2.00 | -2.54 |
| PA1346 | Orn/Lys/Arg decarboxylase N-terminal domain-containing protein | -2.01 | -1.02 |
| PA1214 | asparagine synthetase B | -2.43 | -2.18 |
| plcN | Non-hemolytic phospholipase C, phosphocholine-specific, that hydrolyzes phosphatidylserine and phosphatidylcholine | – | -2.18 |
| PA5196 | ATP-dependent zinc protease | – | -3.03 |

Predicted functions based on sequence homology were searched among available gene and protein databases. Red color indicates values with log2-FC < |1.5|. ‘–‘ indicates not present
